# Parallel multi-droplet platform for reaction kinetics and optimization†

**DOI:** 10.1039/d3sc02082g

**Published:** 2023-08-04

**Authors:** Natalie S. Eyke, Timo N. Schneider, Brooke Jin, Travis Hart, Sebastien Monfette, Joel M. Hawkins, Peter D. Morse, Roger M. Howard, David M. Pfisterer, Kakasaheb Y. Nandiwale, Klavs F. Jensen

**Affiliations:** a Department of Chemical Engineering, Massachusetts Institute of Technology Cambridge MA 02139 USA kfensen@mit.edu; b Pfizer Worldwide Research and Development 445 Eastern Point Rd Groton CT 06340 USA

## Abstract

We present an automated droplet reactor platform possessing parallel reactor channels and a scheduling algorithm that orchestrates all of the parallel hardware operations and ensures droplet integrity as well as overall efficiency. We design and incorporate all of the necessary hardware and software to enable the platform to be used to study both thermal and photochemical reactions. We incorporate a Bayesian optimization algorithm into the control software to enable reaction optimization over both categorical and continuous variables. We demonstrate the capabilities of both the preliminary single-channel and parallelized versions of the platform using a series of model thermal and photochemical reactions. We conduct a series of reaction optimization campaigns and demonstrate rapid acquisition of the data necessary to determine reaction kinetics. The platform is flexible in terms of use case: it can be used either to investigate reaction kinetics or to perform reaction optimization over a wide range of chemical domains.

## Abstract 2

This study develops an automated droplet-based reaction screening platform consisting of ten independent parallel reactor channels. Closed-loop reaction optimizations and a kinetics investigation demonstrate the platform’s capabilities.

## Graphical abstract

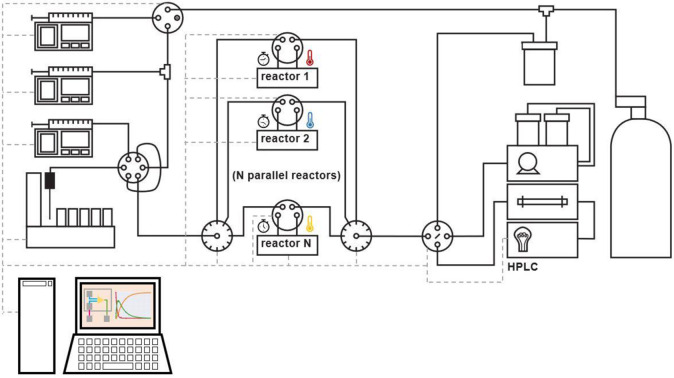

## 1 Introduction

Reaction automation is a key enabling technology that promises to help chemists and engineers explore chemical reaction space in a manner that is both safer and more time- and material-efficient than traditional manual experimentation. A variety of approaches to reaction automation exist,^1^ including well plates with automated dosing and/or sampling^2–5^ and flow reactors based on continuous^6–15^ or stopped-flow^12,16^ operation. Most automated reaction platforms involve an amalgamation of some or all of the following components: liquid- and solid-handling robots to prepare reaction mixtures, pumps to convey fluids to or through either custom-built or commercial reactors, in-line or on-line analytics, and control software to orchestrate all of the operations. Demonstrated applications of fully- or partially-automated reaction platforms span reaction discovery,^2,3,8,17–19^ reaction optimization,^4–6,9–15,20^ reaction characterization,^21–25^ library synthesis,^12,14,16,26^ and compound production.^7,27^

Parallelization has been incorporated into several platforms as a way to increase throughput.^12,14^ When parallelization is coupled with miniaturization, large quantities of reaction information can be generated rapidly using small quantities of material, and the resulting expansion in the design spaces that can be screened is transformative. Platforms that study hundreds to thousands of reactions at a time, each at the nanomole scale, have been reported.^11,28,29^

Efficiency gains can also be realized through the incorporation of iterative optimal experimental design tools into the platform control software,^6,9,10,13,15,30^ particularly when those tools are capable of leveraging preexisting reaction information.^30^ The proliferation of open-source optimization algorithms has made it facile to integrate these into preexisting systems.^31–36^

When the automation objective is not just knowledge generation but also compound synthesis, the ability to automatically perform multi-step synthesis sequences becomes particularly important. A range of approaches have been described for automating multi-step syntheses in ways that retain flexibility, including timed injections of additional reagents into intermediate reaction droplets,^37^ the use of selector valves to create independent reactor channels that allow for the decoupling of subsequent synthesis steps,^12^ and robotic reconfiguration of reactor modules.^15,38^

Despite the incredible progress that has been made, there remains a need for platforms that can reproduce the flexibility, accuracy, and iterative experimental design capabilities of the bench chemist while achieving elevated throughput and using as little material as possible.^25,39^ It is often claimed that reaction automation represents a path toward reduced variability and improved control over reaction outcomes,^40^ but it is important to emphasize that substantial engineering hurdles must be overcome to achieve such fine control, particularly when reactions are being studied at the microscale.

Additionally, it is often cheaper and simpler to design platforms that can be used to study only a subset of chemical reaction space.^41^ For example, many of the platforms that have been developed for conducting high-throughput reaction experimentation were designed with constraints that facilitate the high-throughput nature of the platform while limiting the kinds of chemistries or ranges of operating conditions that can be studied. For example, microfluidic devices constructed using polycarbonate or polydimethylsiloxane (*e.g.* ref. 42) are incompatible with many common organic solvents, and can't operate at high pressures. Other platforms achieve high throughput through the use of well plates, in which all of the reactions on a particular plate are confined to the same temperature and reaction time. Some well plate-based platforms limit reactions to high-boiling solvents and ambient temperature in order to preserve the fidelity of the reactions.^28^ For larger-scale reactions (on the order of a milliliter), well plate-style approaches with glass vials for broad chemical compatibility and a top-plate for sealing can be used to enable heating without solvent loss,^43^ but the confinement of all reactions in the plate to one set of continuous reaction variables remains, at the expense of sampling for reaction kinetic analysis. An alternative approach involves generating unique reaction droplets sequentially in a tube by varying the amount of reagent injected into each droplet,^44^ but the lack of total independence of the conditions of reactions running in parallel persists. To increase the “universality” of automated platforms, various types of automated modular platforms^45^ (some of which autonomously rearrange themselves to suit chemistries and process sequences of interest^38^) have been developed, but there remains a need to translate these concepts of universality to automation at the micro- and nanomole scale.

The platform that we developed is designed to address the aforementioned challenges, and is intended to be used primarily to facilitate efficient reaction development. The platform enables facile exploration of the influence of a variety of categorical and continuous reaction variables on reaction outcomes. For the platform to be useful, it is crucial that the reaction outcomes reported by the platform accurately reflect the reaction conditions specified by the user. The platform combines a bank of parallelized microfluidic reactors that can each individually be controlled across a broad temperature range and operated in either thermal or photochemical mode with a liquid handler and an on-line HPLC. The platform is governed by customized control software that synchronizes all of the hardware and schedules all operations to ensure efficient execution. To meaningfully build on existing capability, at the outset of our design efforts, we set the following goals for performance and operating characteristics of the automated platform:

(1) Excellent reproducibility: <5% standard deviation in reaction outcomes.

(2) Reaction temperatures from 0 to 200 °C (solvent-dependent).

(3) Operating pressure up to 20 atm.

(4) Online analysis: minimal delay between reaction completion and evaluation allows for real-time feedback and eliminates need for quenching and sample stability.

(5) Ability to perform both thermal and photochemical transformations.

(6) Maximally efficient operation through parallelization and scheduling.

(7) Integrated optimal experimental design algorithm for iterative experimentation and optimization.

With all of these capabilities incorporated, our platform fills an important niche. The throughput of our platform is only moderate compared to what can be achieved with nanowell plates, but the conditions in each reactor channel are totally independent, the range of operating conditions is broad, there is flexibility with regard to thermal *versus* photochemical operation, and the inclusion of fully-automated feedback and automated experimental design allows for fully-automated iterative experimentation. Further, prior work on a precursor to this platform showed that the reaction information obtained *via* these droplet reactors is directly scalable.^46^ Thus, the platform is well-suited for situations when it is appropriate to sacrifice maximum throughput in favor of acquiring high-quality data across a wide range of conditions.

Herein, we describe the work we performed to develop our platform and verify that it met all of our design criteria, along with the experiments we conducted to demonstrate the platform's utility.

## 2 Platform design and operation

### 2.1 Design of a parallelized droplet reactor platform for high-fidelity high-throughput reaction screening

The oscillatory droplet flow reactor described by Hwang *et al.*^37^ serves as the foundation for the design of our reactor platform. Microfluidic devices represent one approach to reaction miniaturization: they emerged as a tool for handling and manipulating small quantities of material^47^ and proved to be useful for a wide variety of applications in which samples are precious, including microreactors. However, broad adoption of “flow chemistry” in microreactors was ultimately facilitated by a shift away from traditional microfluidic devices, which are usually specially-fabricated, and toward reactors constructed out of readily-accessible fluoropolymer tubes,^48^ which, like the original devices, feature high surface area to volume ratios that facilitate heat and mass transfer.

To convert the original oscillatory droplet reactor platform to a high-throughput version, we swapped the single reactor channel for a reactor bank consisting of multiple independent parallel reactors. Parallelization allowed us to increase the throughput while retaining flexibility, because each individual reactor can operate at a set of conditions independent of those of its neighbors. We chose to make the parallel channels independent of one another to enable integration with experimental design algorithms, which operate most efficiently when they aren't constrained to propose batches of experiments that have conditions in common. To allow for parallelized droplet oscillation, we initially developed a compact oscillation mechanism, replicates of which could be easily produced and attached to each channel.^49^ The novel mechanism was designed to deliver rapid oscillation for the purposes of efficient mixing, but rapid mixing made solvent loss issues apparent (see Subsection 2.2), which motivated us to switch to stationary operation.

**Fig. 1:**
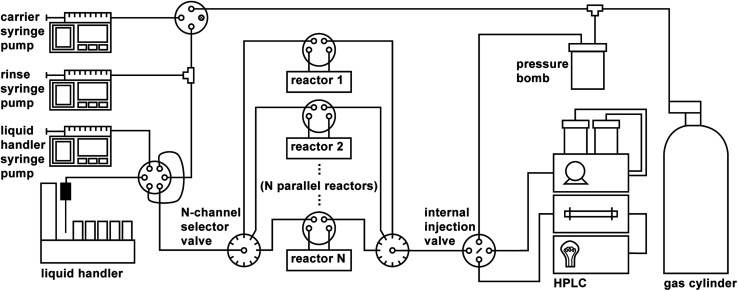
Parallelized platform schematic with *N* parallel stationary reactors.

A schematic of the parallelized platform is shown in Fig. 1. In addition to the components described in ref. 37, the platform features the following additional hardware:

• A reactor bank consisting of a series of parallel reactor channels (we chose to use ten parallel channels by considering the length of bottleneck operations and comparing those to anticipated typical reaction times, see Subsection 3). Selector valves are positioned upstream and downstream of the reactor bank to distribute droplets among the various channels, analogous to the deployment of selector valves in the automated platform developed by Chatterjee *et al.*^12^

• A six-port, two-position valve for each reactor channel that allows each reaction droplet to be isolated from the rest of the system while the reaction is running.

• Two ten-position selector valves (VICI Valco C5H-3720EUHAY) upstream and downstream of the reactor bank that allow for distribution of droplets to their assigned reactors, and collection of droplets from the reactor bank for analysis.

• An internal injection valve (VICI Valco C84H-1574-.02EUHA) for sampling droplets for HPLC analysis, whose swappable nanoliter-scale rotors (20 nL, 50 nL, 100 nL) enable minuscule injection volumes that (1) eliminate the need to dilute concentrated reactions prior to analysis and (2) mitigate the effects of strong solvents on the analytical outcome.

### 2.2 Development and validation of a single-channel reactor prototype

As a precursor to parallelization, we aimed to demonstrate satisfactory performance against the criteria outlined in Section 1 using a single-channel version of the platform.

One of the criteria we emphasized while designing the platform was reproducibility. Every step in the process that influences reaction outcomes can also impact reproducibility. Some of these factors require simple calibration and standardization, such as ensuring that each thermocouple is calibrated and positioned in the same location on the reactor plate; other factors are intrinsic features of equipment that was incorporated into the platform as-is, such as the consistency of the HPLC injection. To prevent carryover between droplets from impacting reproducibility, we incorporated multiple rinse droplets between each droplet transit operation.

The Gilson liquid handler that we used to prepare reaction droplets allows for extensive customization of factors that can have a significant influence on reproducibility. To ensure fidelity to user-specified concentrations, we optimized the liquid handler design and operation parameters to deliver deviations from target concentrations of <5% (see ESI Section S3.2†). We expect these experiments to be of interest to automated experimentation scientists generally, given the widespread use of liquid handlers in automated systems.

**Fig. 2:**
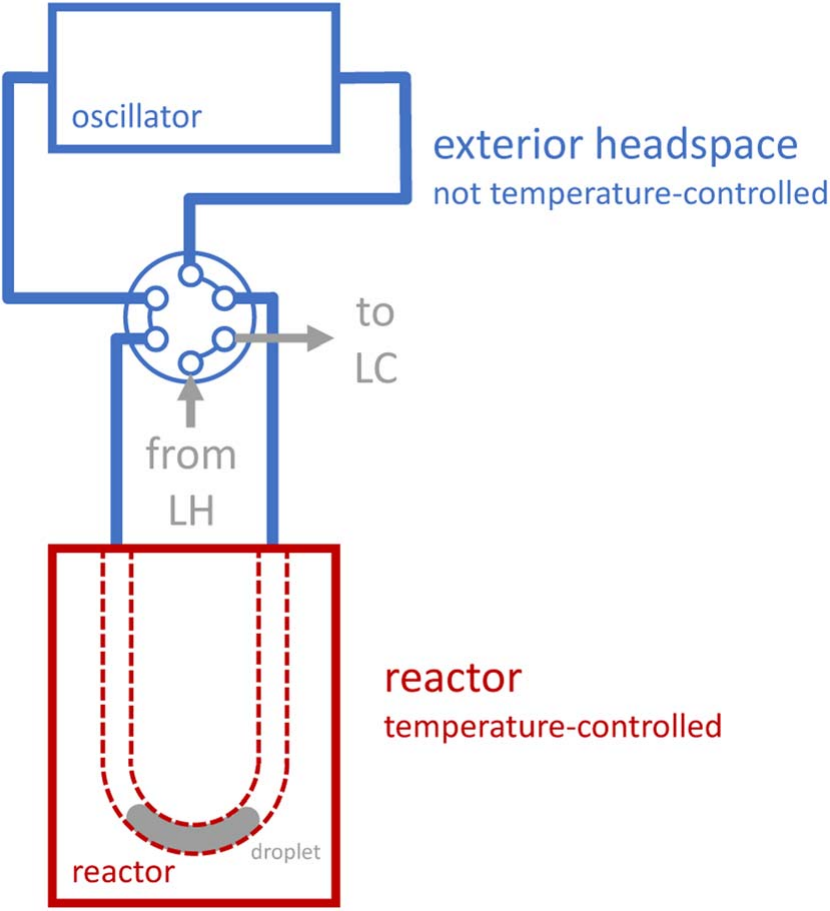
Temperature control of droplet and droplet headspace. The blue region is not explicitly temperature-controlled. The red region corresponds to the aluminum plates that sandwich the reactor, which can be both heated and cooled to match user- or algorithm-specified temperature. LH: liquid handler. LC: liquid chromatography (HPLC).

Reaction execution also requires special consideration. During our early development experiments with the novel oscillator, we observed significant evaporation effects as reactions proceeded: reaction droplets would shrink over time, and corresponding droplets of condensed liquid would grow outside the reactor. Unlike a reaction in a sealed, jacketed vessel, the droplet does not merely evaporate to saturate the headspace, and then cease evaporating once equilibrium is reached. Rather, several distinct phenomena contribute to a more dynamic behavior, in which volatile components in the droplet evaporate to saturate the headspace, the vapor which is saturated at the elevated reactor temperature exits the heated reactor (due to both convection from the induced oscillation and diffusion, see ESI Section S4†), the vapor then becomes supersaturated in the cooler tubing outside the reactor (which is not temperature-controlled, see Fig. 2), and condenses, thus forcing further evaporation of the droplet to maintain saturation of the heated headspace inside the reactor. To provide a more quantitative picture of evaporation effects, we measured the apparent charge error for a series of preliminary reactions that we performed at a range of temperatures and residence times using the oscillatory reactor (see ESI Section S4†). The apparent charge error values skew positive (corresponding to “overcharge”), with an average value of 70%, which is consistent with significant evaporation effects.

Based on our assessment of the driving forces for evaporation and our experimental observations of its significant impact on data quality and reproducibility, we determined that rapid oscillation of the reaction droplets is fundamentally at odds with another goal of the platform, which is to study reaction droplets at high temperatures (including temperatures that exceed the atmospheric boiling point of the solvent) without limits on the residence time. To remain faithful to our high-fidelity criterion and retain compatibility with high temperatures and essentially unlimited residence times, we eliminated the oscillatory component from the original platform design, which helps mitigate evaporation, but requires us to constrain our reaction space to those reactions which are homogeneous initially. We considered this preferable to using exclusively high-boiling solvents which would have further limited reaction scope.^28^ Accordingly, we redeveloped the reactor for “stationary” operation with acceptable restrictions on reaction scope; the resulting reaction operation style is similar to the “stopped-flow” approach described by both Chatterjee *et al.*^12^ and Avila *et al.*^16^

We designed a new single-channel reactor that uses tubing with a small (0.02′′) inner diameter to minimize the interfacial area between the gas and the liquid and therefore slow evaporative behavior. We also minimized the total reactor headspace to minimize solvent loss due to vapor-liquid equilibrium; we determined an appropriate total volume of tubing by accounting for thermal expansion and gas evolution from a model decarboxylation reaction (see ESI Section S1†).

**Fig. 3:**
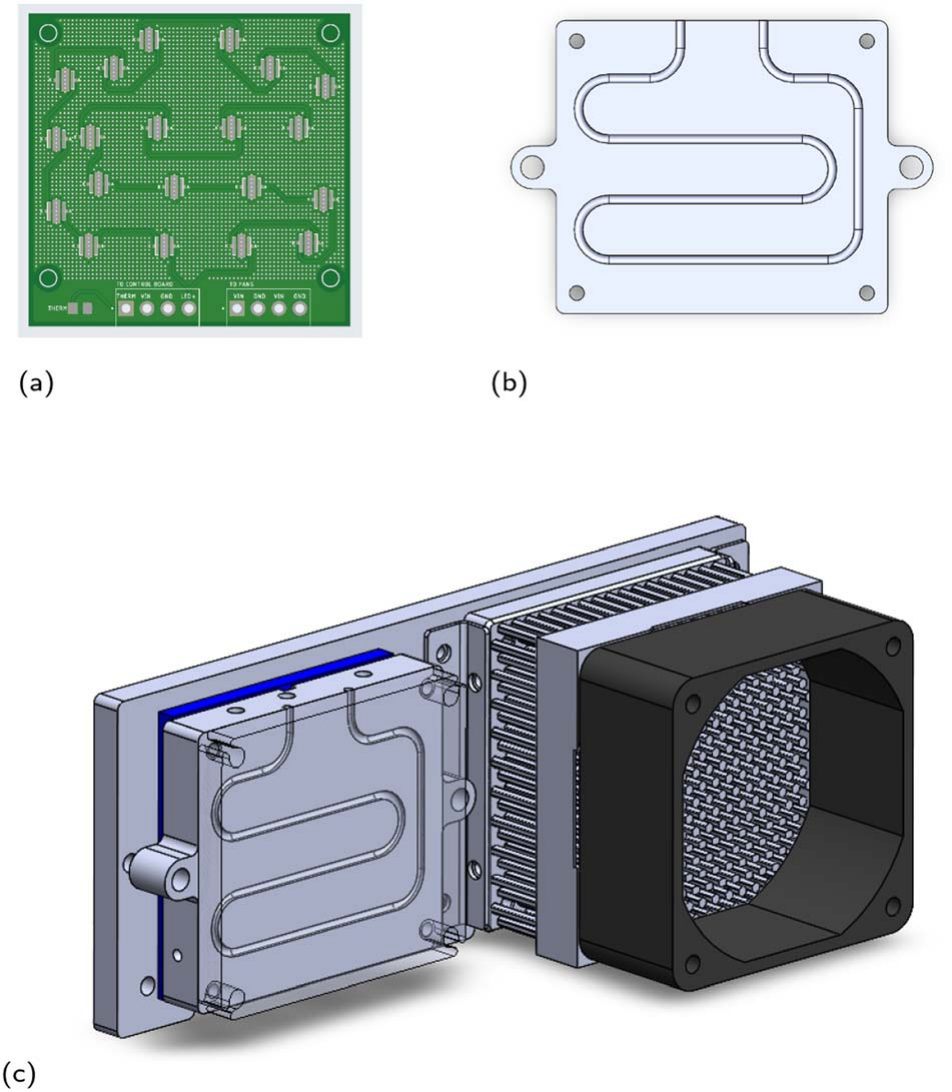
SolidWorks sketches of the photochemistry hardware. (a) LED board with LED layout corresponding to the stationary reactor. (b) Stationary reactor tubing path. (c) SolidWorks sketch of the reactor, Peltier module, heat sink, and fan housing.

The updated stationary reactor that accounts for these conditions with safety factors has a heated volume of 60 μL and connects to the six-port valve *via* eight total inches of temperature-uncontrolled 0.02′′ ID FEP tubing, which translates to a volume of about 40 μL. A SolidWorks sketch of the reactor is included in Fig. 3b. Evaporation studies verified that zero measurable evaporative losses from a droplet of pure THF are observed over 30 min at 125 °C (see ESI Section S4†). The droplets do expand upon heating, but we did not observe any translation of the droplets that could be due, for example, to thermocapillary effects, since there is little to no temperature gradient within the reactor itself (see ESI Section S4†).

**Scheme 1:**
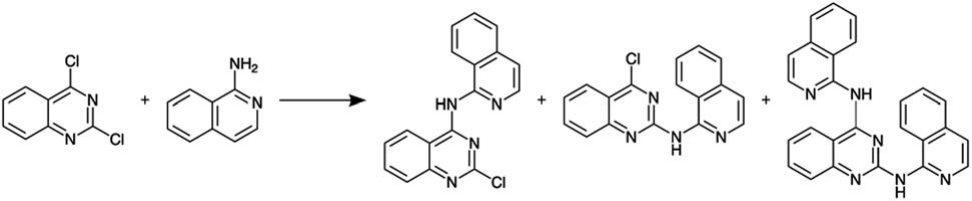
Nucleophilic aromatic substitution reaction used to validate the single-channel version of the platform.

Before constructing the full parallelized reactor, we performed preliminary demonstration experiments using a model homogeneous nucleophilic aromatic substitution (S_N_Ar) reaction (Scheme 1) that motivated additional modifications to our droplet handling procedure (see ESI Section S3.2 for additional details†). After the additional modifications were incorporated, we performed a final set of validation experiments using the same model reaction. The final validation experiments indicated excellent reproducibility (see ESI Section S3.2†).

#### 2.2.1 Adaptation of the single-channel prototype for photochemical operation

To make the platform compatible with photochemical reactions, we designed a circuit board containing a series of light-emitting diodes (LEDs) that can be placed facing the reactor. We chose to pattern-match the LEDs to the stationary reactor path in order to deliver as many photons as possible to the reaction mixture with the smallest number of LEDs. Minimizing the quantity of LEDs makes it easier to keep both the reactor and the LED board itself cool. A schematic of the LED board is shown in Fig. 3a.

A common photoreactor design challenge is temperature control. To keep the reactor cool while the LEDs are operational, a Peltier module was incorporated into the design: a Peltier device (Multicomp Pro part number MCPE-241-14-15) was sandwiched between the stationary reactor and a backboard using thermal paste, with the “cold side” of the Peltier touching the reactor. On the opposite end of the backboard, we mounted a heat sink (Alpha Novatech part number FH6030MU) and fan to remove the heat generated on the “hot side” of the Peltier and maintain its efficiency. A 3D sketch of the module is provided in Fig. 3c.

To supply power and control to the Peltier module, LED board, and heat sink fans, we designed and constructed a custom circuit board (see ESI Section S1.2†). The LabVIEW-based platform control software communicates with the circuit boards *via* a Python node.

To validate the platform's photochemical capabilities, we sought to:

(1) Define an absolute minimum separation distance between the reactor and the LED board that was large enough for the Peltier cooler to maintain the reactor temperature below 25 °C when the LEDs are operated at full power;

(2) Further define an appropriate separation distance (equal to or greater than the temperature-based distance) that delivered sufficiently uniform radiation to the reactor; and

(3) Affirm that the performance was consistent across three identical copies of the LED board, to validate readiness for parallelization.

For the purposes of these initial tests, three identical boards were sent for production at Bittele‡‡https://www.7pcb.com using 455 nm LEDs (Cree LED part number XTEARY-00-0000-000000N02). We started by determining the minimum distance between the LED board and the reactor (see ESI Section S2†).

**Scheme 2:**
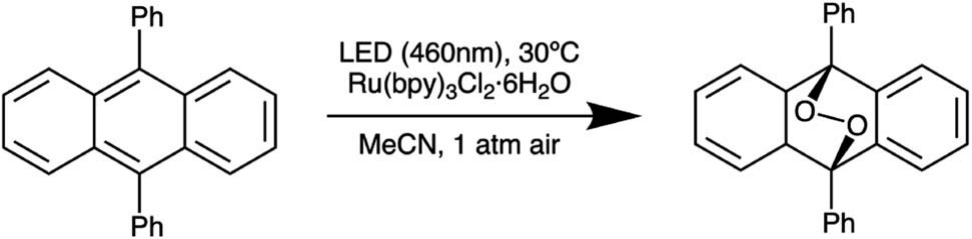
Aerobic oxidation of 9,10-diphenylanthracene to the corresponding peroxide in the presence of Ru(bpy)_3_Cl_2_ used to validate irradiance uniformity.

Next, we further optimized the distance between the reactor and LED board on the basis of irradiance uniformity by performing five different 9,10-diphenylanthracene (DPA) oxidation reactions (Scheme 2) at each of a series of separation distances, while allowing the natural variation in droplet placement in the reactor to provide a representative assessment of the performance at different locations within the reactor (Table 1). Since the absolute irradiance reaching the reaction mixture decreases as distance from the LED board increases, we increased the residence time of the droplets in parallel with the increasing separation distance in order to maintain comparable conversion values (approximately 50% in each case) across the various separation distances. Based on the results in Table 1, the reaction is fairly reproducible across the range of separation distances we tested. At the minimum separation distance of 2 cm, the standard deviation in final DPA concentration is slightly elevated compared to the rest of the values, suggesting greater nonuniformity than the cases where the reactor and LED board are further apart. On the basis of these results, we chose to fix the reactor-board separation distance at 4 cm.

**Table 1:** Results of irradiance uniformity assessment. Five reactions were performed at each separation distance. The residence time was increased with the increasing separation distance so as to deliver final conversion values of approximately 50% at each distance

Distance^a^ [cm]	Residence time [s]	Standard deviation in final DPA concentration [%]
2	40	3.9
3	60	1.6
4	65	2.6
5	70	1.5
6	90	1.2

a Distance between reactor and LED board.

**Scheme 3:**
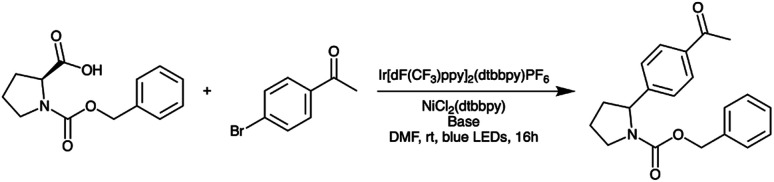
Model photoredox reaction used to verify that different LED boards behave similarly.

Finally, we used a model photoredox reaction (Scheme 3) to verify that the three LED boards we ordered behave similarly. For each board, we performed one photoredox reaction at each of two different power levels. Then, for each power level, we computed the standard deviation in conversion values across the three boards to assess cross-board variation (Table 2). As a baseline for the variation assessment, we also performed three photoredox reactions at the higher of the two power levels (39%) using LED board #1. The standard deviation in conversion across these three experiments was 3.1%, which is similar to the degree of variation observed across the boards, confirming that the boards behave very similarly.

**Table 2:** Results of LED board variation assessment: conversion values for the model photoredox reaction depicted in Scheme 3 at two different LED power levels for each of three different LED boards. Conversion was measured after ten minutes in the illuminated reactor maintained at 30 °C

LED power [%]	Board #1 [% conversion]	Board #2 [% conversion]	Board #3 [% conversion]	Standard deviation [%]
39	55.6	58.5	52.4	3.1
12	27.1	30.9	25.8	2.7

## 3 Software for scheduling parallel operation and reaction optimization

### 3.1 Design of the scheduling algorithm

**Fig. 4:**
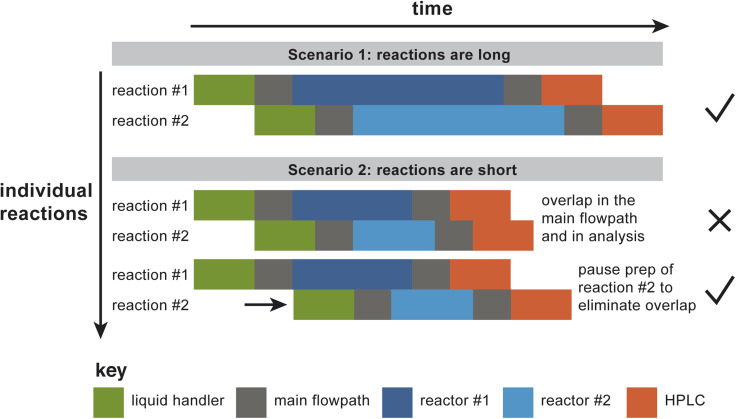
Demonstration of the need for a scheduling operation. The reactors are bookended by bottleneck operations that only one reaction droplet can occupy at one time: the liquid handling operation (represented in green in the figure), transit form the liquid handler to the target reactor and from the target reactor to the HPLC (both of which occupy the main flowpath, shown in gray), and the HPLC itself (represented in orange in the figure). In scenario 1, the reactions – the only parallelized aspect of the platform – are long relative to the bottleneck operations. Under these circumstances, it works to run the droplet preparation steps back-to-back. In scenario 2, the reactions are relatively short; here, droplet preparation cannot be back-to-back without collisions. By pausing the droplet preparation until an appropriate time, collisions are avoided.

In order to take advantage of the parallelized hardware, the software must define a schedule that determines the time at which each reaction droplet must be subjected to each of a series of predefined hardware operations. As currently constructed, the platform has bottlenecks upstream and downstream of the reactor bank, for droplet preparation (*via* a liquid handler) and analysis (by HPLC). To decide when to start preparing a particular reaction droplet, the software must account for preexisting demands on the liquid handler, transport in the main flowpath, the rinse and venting operations, the reactors, and the analytical system. Without advance planning, the naive operation mode would be to prepare reaction droplets back-to-back, which could result in “collisions” at different points in the process, where a “collision” is defined broadly as any situation where distinct droplets are occupying the same section of hardware at the same time. Fig. 4 illustrates the situation more explicitly, by showing the time course of two reaction droplets to be studied using two parallel reactors. In the first case illustrated in the figure, the reactions are long relative to the other operations in the process, so making the default assumption that the two different droplets can be prepared back-to-back works (*i.e.*, there are no collisions). In the second illustrated case in Fig. 4, the reactions are relatively short; here, preparation of the second droplet must be paused until it can be prepared in such a way that guarantees that bottleneck operations won't overlap.

The reason that “collisions” as defined broadly above must be avoided is twofold: first, under collision conditions, droplets won't necessarily merge, but they might; second, it would be difficult to design a tracking system that would be capable of confidently distinguishing between the droplets. Therefore, the surest way to avoid errors is to design the scheduling algorithm such that no more than one droplet is expected to occupy a given section of hardware at any one time. The scheduler must satisfy the following requirements:

• Once prepared, a droplet must be immediately transported to its assigned reactor to minimize the impact of any room-temperature reactivity on the outcome.

• For the same reason listed in the bullet above, once a droplet has been confined to its reactor for its specified residence time, it must be immediately transported to analysis.

• All droplet transit operations must be immediately followed by a rinse/vent operation to prevent carryover to subsequent droplets. Two types of rinse and vent operations are necessary: one that does not include a reactor in the flowpath, which follows droplet transit from the liquid handler injection valve to its reactor; and another that follows droplet transit from the reactor to the analytical sampling valve, and which therefore does rinse the reactor in question as well.

Initially, we considered ways to design the scheduling algorithm such that it produced a schedule *a priori* based on user-provided operation times for the liquid handler, both transit operations, both rinse/vent operations, the analytical method, and of course the reactions themselves, but there are too many possible cases to construct the scheduler in that manner. Instead, we wrote the scheduler so that it starts by assuming that droplet preparation can be back-to-back. Then, the scheduler scans through the reactions in order of execution, looking for cases where droplets would collide (as defined above). If collisions are detected, the algorithm begins iteratively shifting the preparation of the first colliding droplet back by one unit of time until all collisions are eliminated. The algorithm is written in MATLAB, and it is very fast: it can schedule 500 reactions in less than a tenth of a second. See ESI Section S3† for additional details.

Scheduling is one important focus of the rich field of operations research, which emphasizes operational efficiency. Two quantitative measures of the efficiency of a process are total operation time (which is, ideally, minimized) and utilization (ideally maximized). The parallelized droplet reactor is a version of a classic scheduling problem in which *m* unique jobs are to be scheduled on *n* identical parallel machines and the objective is to minimize the “makespan”, or total time necessary to complete all *m* jobs. Minimizing the makespan in this case is complex; an early approximation to a universal solution is known as the Longest Processing Time (LPT) rule,^50^ in which the *m* jobs are sorted from longest to shortest (in our case, from the longest target residence time to the shortest) and run in that order. There are alternatives to LPT that improve on its performance in edge cases (see, *e.g.* ref. 51), but LPT remains a popular heuristic for its simplicity and short run time. We implemented LPT in the scheduling algorithm in order to approximately minimize the total run time of a given set of reactions.

The scheduling algorithm does not directly account for reactor heating or cooling rates; instead, the control software pauses droplet production until the temperature of the droplet's assigned reactor has reached its target value. A more direct accounting of the heating and cooling rates by the scheduling algorithm would increase overall efficiency. Heating operations – which rely on cartridge heaters – are quite rapid compared to the time necessary to prepare a reaction droplet, but cooling can take several minutes, depending on the magnitude of the gradient and the initial temperature of the heat sink.

## 4 Demonstration campaigns

To showcase the utility of the fully-parallelized platform diagrammed in Fig. 1 with ten parallel reactors, we performed two distinct types of experimental campaigns: (1) a reaction kinetic analysis and (2) a series of closed-loop optimization campaigns. Both of these types of investigations depend on very high-quality data and benefit from the acceleration enabled by parallelized execution, a combination that our platform uniquely offers.

### 4.1 Time- and material-efficient kinetics investigation

Acquiring the data necessary to characterize the kinetics of a reaction can be laborious. When reactions are studied in traditional batch mode, many reactions must in general be performed to determine the reaction rate law(s) and the temperature dependence of the rate constant(s). Further, reaction environments aren't always amenable to sampling over time; in these cases, separate reactions have to be executed to evaluate each timepoint of interest. Thanks to our platform's miniaturized-and-parallelized format, the platform offers the ability to perform kinetic investigations in a time- and material-efficient manner, thus accelerating reaction evaluation and understanding.

**Scheme 4:**
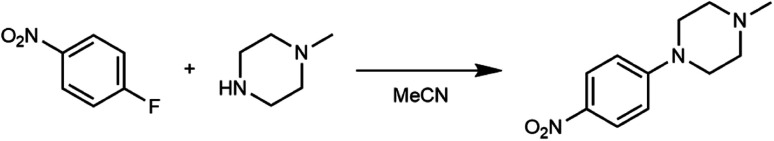
Reaction used to demonstrate the accurate and rapid determination of kinetic parameters on the platform.

To showcase the benefits of collecting kinetic data on our platform, we sought to reproduce the results of a preexisting kinetics study^52^ examining a nucleophilic aromatic substitution reaction between 4-fluoronitrobenzene and 1-methylpiperazine in acetonitrile as solvent (Scheme 4). First, we verified the rate law determined by Ashworth *et al.* (eqn (1)) by performing the reaction at 70 °C in the presence of two different concentrations of the aryl halide (the limiting reagent) and tracking the consumption of the aryl halide. We took advantage of the ten parallel reactors to rapidly evaluate both conditions at residence times of 5, 10, 15, 25 and 40 minutes. Next, we measured the temperature dependence of the rate constant by studying the reaction at 60, 80, and 90 °C. Note that 90 °C exceeds the atmospheric boiling point of the solvent, which is accessible thanks to the platform's unique pressurized design.

1 *r* = *k*[ArF][R_2_NH]^2^

We used the MATLAB functions *nlinfit* and *ode45* to determine the rate constants at each temperature, and then fitted the data to the Eyring equation (eqn (2)) to determine the enthalpy and entropy of activation of the reaction for comparison to the literature.^52^2
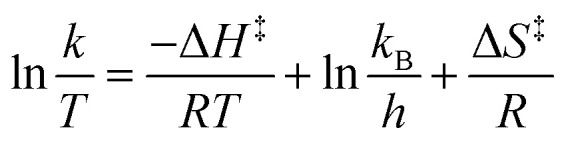


**Fig. 5:**
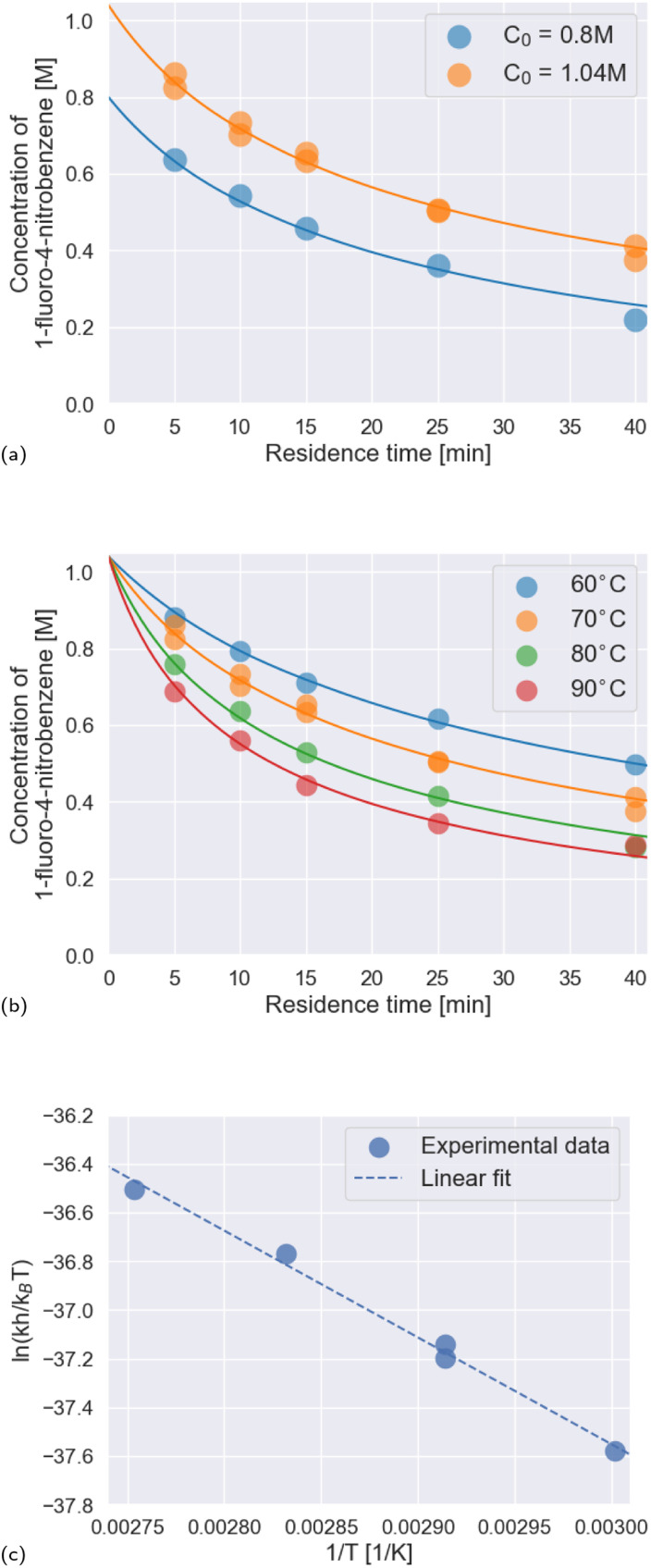
Results of the kinetics investigation. (a) Temporal reaction profiles at 70 °C for two different initial concentrations of the limiting reagent, 1-fluoro-4-nitrobenzene: 0.8 M and 1.04 M. Scatter points represent experimental data; line plots represent the kinetic model. (b) Temporal reaction profiles at temperatures from 60 °C to 90 °C in ten-degree increments, with the initial concentration of the limiting reagent, 1-fluoro-4-nitrobenzene, fixed at 1.04 M. Scatter points represent experimental data; line plots represent the kinetic model. (c) Eyring plot for measured rate constants. Linear regression of the experimental data yielded eqn (3), which is plotted with a dashed line in the figure.

To robustly determine kinetic parameters for the reaction, we performed a total of thirty reactions, which required just thirteen hours of platform time and a combined 600 mg of the two starting materials. We observed excellent agreement between the rate law and rate constants determined by Ashworth *et al.* and those that we measured (Fig. 5a and b and Table 3).

**Table 3:** Comparison between the rate constants we measured and literature values. Specified uncertainties correspond to standard errors

Temperature	*k* [M^−2^ s^−1^] × 10^4^	Literature values^52^
60 °C	3.32 ± 0.07	2.87, 3.03
70 °C	5.00 ± 0.14	5.10, 5.15, 5.08
80 °C	7.90 ± 0.30	7.77, 7.75
90 °C	10.6 ± 0.40	—

We constructed an Eyring plot (Fig. 5c) to determine the enthalpy and entropy of activation of the reaction. Linear regression of the experimental data yielded the following equation:3
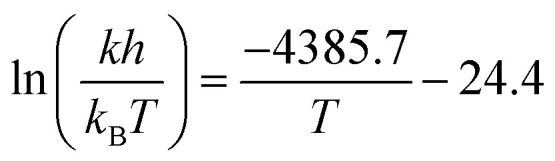


The linear fit was used to determine the following enthalpy and entropy of activation: Δ*H*^‡^ = 36.5 ± 1.9 kJ mol^−1^ and Δ*S*^‡^ = −203 ± 5 J mol^−1^ K^−1^. The thermodynamic values we determined differ somewhat from the literature values of Δ*H*^‡^ = 44.5 kJ mol^−1^ and Δ*S*^‡^ = −180 J mol^−1^ K^−1^,^52^ due in part to the fact that we explored a wider temperature range than Ashworth *et al.* When the thermodynamic constants are recomputed without the 90 °C data, the values are closer to those previously reported in the literature: Δ*H*^‡^ = 39.5 kJ mol^−1^ and Δ*S*^‡^ = −194 J mol^−1^ K^−1^.

### 4.2 Optimal experimental design with Bayesian optimization routine Dragonfly

To perform automated, closed-loop reaction optimization on our platform, we integrated the open source Bayesian optimization package Dragonfly^53^ with our control software *via* a Python node. Bayesian optimization is a framework for iterative global black-box derivative-free optimization^54^ that relies on a probabilistic surrogate model (Gaussian processes) combined with one of a variety of acquisition functions^55^ that determine which experiments to perform next by balancing exploitation of regions expected to offer optimal values of the objective function with exploration of regions where the uncertainty of the surrogate model is high. Bayesian optimization has been successfully applied to a variety of reaction optimization problems.^15,31–36,56^

We decided to deploy the Dragonfly implementation of Bayesian optimization for several reasons. One of the advantages of the Dragonfly package is that it avoids performance artefacts that are specific to a single acquisition function by adaptively sampling from a set of popular acquisition functions (upper confidence bound, expected improvement, and Thompson sampling) in a manner that progressively prefers acquisition functions that perform better on the problem in question.^53^ Unlike a popular, reaction-specific option (EDBO^35^ and EDBO+^36^), Dragonfly explicitly optimizes the acquisition function to select the next point(s) (using DiRect, PDOO, or evolutionary algorithms, automatically selected by Dragonfly depending on the nature and dimensionality of the domain). Further, Dragonfly allows for multiobjective optimization and for a wide variety of variable types (continuous, discrete numeric, and discrete categorical).

We applied Dragonfly to a series of reaction optimization case studies, each consisting of both continuous and categorical optimization variables. We operated Dragonfly in ask-tell mode with primarily default settings (see ESI Section S5.0.1 for details†). We used one-hot encoding to distinguish between the various settings of each categorical variable. The optimization objective in each case study was product peak area determined by HPLC, normalized by the peak area of an internal standard. Algorithm termination was based on manual assessment of convergence.

**Scheme 5:**
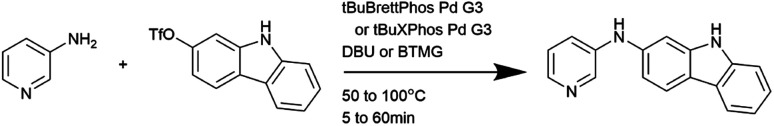
Reaction used to showcase automated closed-loop optimization on the platform.

We used Dragonfly to perform closed-loop optimization of the Buchwald–Hartwig amination between 9*H*-carbazol-2-yl trifluoromethanesulfonate and 3-aminopyridine (Scheme 5) over catalyst (tBuBrettPhos Pd G3 or tBuXPhos Pd G3), base (DBU or BTMG), temperature (50 to 100 °C) and residence time (5 to 60 minutes). We performed the optimization campaign twice, once in dimethylformamide (DMF) and once in dimethyl sulfoxide (DMSO), to examine the influence of solvent on reaction performance. In both solvents, an optimum was identified within a few dozen experiments (ESI Fig. S21a and b†); for the DMF campaign, 28 experiments were performed in 12.5 hours, consuming 132 mg of the aryl triflate, and for the DMSO campaign, 30 experiments were performed over 14 hours, requiring the consumption of 142 mg of aryl triflate.

**Fig. 6:**
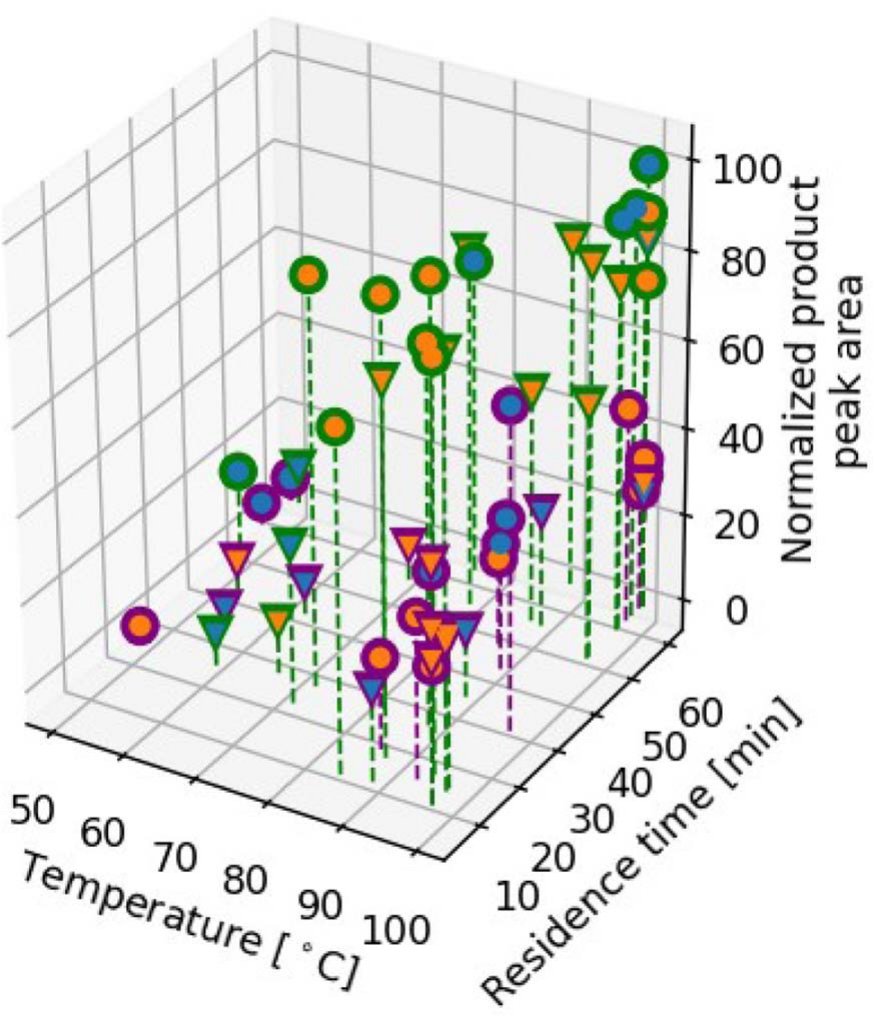
Optimization results in DMF and DMSO. Marker shapes denote the identity of the base: circles for DBU and triangles for BTMG. Marker colors denote the identity of the catalyst and solvent: green denotes DMF, purple denotes DMSO, blue denotes tBuBrettPhos Pd G3, and orange denotes tBuXPhos Pd G3.

When the reaction is performed in DMF, many conditions are found that deliver nearly full conversion: a quarter of the reactions that were performed during the campaign had outcomes that came within ten percent of the optimum. In contrast, when the reaction is performed in DMSO, high-performing conditions are more difficult for the algorithm to find, and the optimal objective function value that the algorithm identified is 30% lower than the optimum identified in DMF. The relatively poor performance of this reaction in DMSO compared to its performance in DMF highlights the importance of platforms that enable screening of solvents other than DMSO (Fig. 6).

Across both campaigns, the best-performing catalyst–base combination is tBuBrettPhos Pd G3 and DBU, and the best performance is observed at the maximum temperature in the range examined. Residence time was not found to have a large effect on reaction outcomes in either solvent, despite the fact that we examined a wide residence time range; high-conversion conditions could be identified even at the low end of the residence time range.

**Scheme 6:**
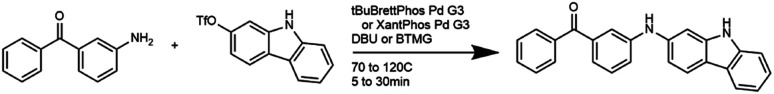
Reaction used to showcase automated closed-loop optimization on the platform.

We separately optimized another Buchwald–Hartwig amination with 9*H*-carbazol-2-yl trifluoromethanesulfonate, this time using tetrahydrofuran (THF) as the solvent and (3-aminophenyl)(phenyl)methanone as the amine (Scheme 6). We optimized this reaction over catalyst (tBuBrettPhos Pd G3 or XantPhos Pd G3), base (DBU or BTMG), temperature (70 to 120 °C) and residence time (5 to 30 minutes). For this reaction, the XantPhos precatalyst yielded little to no product, whereas tBuBrettPhos resulted in full conversion at the high end of the temperature range, regardless of the residence time. This campaign was completed in 28 experiments, which required ten hours of execution time and the consumption of 132 mg of aryl triflate.

The successful use of THF in the second optimization campaign highlights the platform's compatibility with relatively volatile solvents. DMSO is commonly used as a solvent in high-throughput reaction screening platforms because its low vapor pressure and high boiling point help ensure the fidelity of reaction concentrations and because it easily dissolves a wide variety of compounds, but its high solvation tendency poses challenges when processes are later scaled and optimized for isolated yield and safety. Further, the performance of the first optimization campaign in DMSO highlights the significance of solvent effects and motivates the design and implementation of platforms like ours, which can screen reactions in more process-realistic solvents.

## 5 Conclusions

The platform we developed allows for the rapid generation of high-quality reaction data. We sought to make the platform as flexible as possible, thereby minimizing the restrictions imposed on the design space by the scope of capabilities of the platform. Applications of our platform include reaction screening and optimization activities that are part of typical process development efforts, as well as data generation to support chemical machine learning. Through the incorporation of a fraction collector, the platform could also be used for compound synthesis to support medicinal chemistry programs.

There are important challenges that remain to be addressed. The throughput of our platform is only moderate, and could be improved through the incorporation of tandem bottleneck instruments as well as shorter analytical methods and shorter liquid handler processing times. Fast, accurate, affordable, and easy-to-use solids-handling equipment could have eliminated the time-consuming manual effort required to prepare stock solutions for the liquid handler. Efficiency gains could also be realized through further optimization of the scheduling algorithm to account for reactor heating and cooling rates.

Additionally, fully-automated analysis is a persistent challenge. Ideally, it would be straightforward to determine the yield of each product of every reaction, but yield determination requires calibration, which in turn requires authentic product standards. Internal standards can be useful to allow for simple normalized response as output rather than yield (the approach we used here). The responses of the various reaction components must be distinguishable from one another in the chosen analytical approach. The recently-reported MOCCA software (and other previous efforts)^57^ is helping on this front in the U/HPLC space: MOCCA uses the full UV-Vis spectra recorded by diode array detectors at each timepoint, which enables deconvolution of overlapping peaks. Automated analysis could also be facilitated through the incorporation of automatic response factor estimation.^58^

To improve the efficiency of the reaction optimization protocol, robust approaches to defining batches of experiments that can be performed in parallel are needed. In general, performing batches of experiments from an iterative experimental design routine on a parallelized platform involves a tradeoff between rapid collection of additional data (which is gained through parallel operation) and the possibility that the experiments in the batch are redundant. Thompson sampling represents one approach to batched Bayesian optimization that has been found to perform well in a variety of reaction optimization scenarios.^32,59,60^

## Data availability

All experimental data described are available in the main text or the ESI.† The platform control software is available in the following GitHub repository: https://github.com/natalieeyke/parallel-droplet-platform.

## Author contributions

NSE, JMH, SM and KFJ formulated the project. NSE and KFJ designed the system and NSE constructed the system with the help of BJ and TH. NSE developed the software. NSE and TNS performed the experiments and analyzed the results. SM, JMH, PDM, RMH, DMP, KYN, and KFJ provided feedback and guidance on system design, operation, and chemistry selection. NSE wrote the initial manuscript. NSE, TNS, SM, RMH, and KFJ edited the manuscript, and all authors reviewed the manuscript. SM and KFJ supervised the project, and JMH, SM, and KFJ secured the funding.

## Conflicts of interest

There are no conflicts to declare.

## Supplementary Material

SC-014-D3SC02082G-s001

## References

[cit1] Mennen S. M., Alhambra C., Allen C. L., Barberis M., Berritt S., Brandt T. A., Campbell A. D., Castañón J., Cherney A. H., Christensen M., Damon D. B., de Diego J. E., Garcia-Cerrada S., Garcia-Losada P., Haro R., Janey J., Leitch D. C., Li L., Liu F., Lobben P. C., MacMillan D. W. C., Magano J., McInturff E., Monfette S., Post R. J., Schultz D., Sitter B. J., Stevens J. M., Strambeanu I. I., Twilton J., Wang K., Zajac M. A. (2019). Org. Process Res. Dev..

[cit2] Dreher S. D., Dormer P. G., Sandrock D. L., Molander G. A. (2008). J. Am. Chem. Soc..

[cit3] McNally A., Prier C. K., MacMillan D. W. (2011). Science.

[cit4] Metz A. E., Berritt S., Dreher S. D., Kozlowski M. C. (2012). Org. Lett..

[cit5] Tu N. P., Dombrowski A. W., Goshu G. M., Vasudevan A., Djuric S. W., Wang Y. (2019). Angew. Chem..

[cit6] McMullen J. P., Jensen K. F. (2010). Annu. Rev. Anal. Chem..

[cit7] Ingham R. J., Battilocchio C., Hawkins J. M., Ley S. V. (2014). Beilstein J. Org. Chem..

[cit8] Sun A. C., Steyer D. J., Allen A. R., Payne E. M., Kennedy R. T., Stephenson C. R. (2020). Nat. Commun..

[cit9] Reizman B. J., Jensen K. F. (2016). Acc. Chem. Res..

[cit10] Fitzpatrick D. E., Battilocchio C., Ley S. V. (2016). Org. Process Res. Dev..

[cit11] Perera D., Tucker J. W., Brahmbhatt S., Helal C. J., Chong A., Farrell W., Richardson P., Sach N. W. (2018). Science.

[cit12] Chatterjee S., Guidi M., Seeberger P. H., Gilmore K. (2020). Nature.

[cit13] Jeraal M. I., Sung S., Lapkin A. A. (2021). Chem.: Methods.

[cit14] Ahn G.-N., Sharma B. M., Lahore S., Yim S.-J., Vidyacharan S., Kim D.-P. (2021). Commun. Chem..

[cit15] Nambiar A. M., Breen C. P., Hart T., Kulesza T., Jamison T. F., Jensen K. F. (2022). ACS Cent. Sci..

[cit16] Avila C., Cassani C., Kogej T., Mazuela J., Sarda S., Clayton A. D., Kossenjans M., Green C. P., Bourne R. A. (2022). Chem. Sci..

[cit17] Shevlin M., Friedfeld M. R., Sheng H., Pierson N. A., Hoyt J. M., Campeau L.-C., Chirik P. J. (2016). J. Am. Chem. Soc..

[cit18] Vandavasi J. K., Hua X., Halima H. B., Newman S. G. (2017). Angew. Chem., Int. Ed..

[cit19] Bayly A. A., McDonald B. R., Mrksich M., Scheidt K. A. (2020). Proc. Natl. Acad. Sci. U. S. A..

[cit20] Burgess K., Lim H.-J., Porte A. M., Sulikowski G. A. (1996). Angew. Chem., Int. Ed. Engl..

[cit21] Mozharov S., Nordon A., Littlejohn D., Wiles C., Watts P., Dallin P., Girkin J. M. (2011). J. Am. Chem. Soc..

[cit22] Moore J. S., Jensen K. F. (2014). Angew. Chem..

[cit23] Nunn C., DiPietro A., Hodnett N., Sun P., Wells K. M. (2018). Org. Process Res. Dev..

[cit24] Aroh K. C., Jensen K. F. (2018). React. Chem. Eng..

[cit25] Li X., Dunn A. L. (2021). Org. Process Res. Dev..

[cit26] Li J., Ballmer S. G., Gillis E. P., Fujii S., Schmidt M. J., Palazzolo A. M., Lehmann J. W., Morehouse G. F., Burke M. D. (2015). Science.

[cit27] Adamo A., Beingessner R. L., Behnam M., Chen J., Jamison T. F., Jensen K. F., Monbaliu J.-C. M., Myerson A. S., Revalor E. M., Snead D. R. (2016). et al.. Science.

[cit28] Buitrago Santanilla A., Regalado E. L., Pereira T., Shevlin M., Bateman K., Campeau L.-C., Schneeweis J., Berritt S., Shi Z.-C., Nantermet P., Liu Y., Helmy R., Welch C. J., Vachal P., Davis I. W., Cernak T., Dreher S. D. (2015). Science.

[cit29] Jaman Z., Logsdon D. L., Szilagyi B., Sobreira T. J., Aremu D., Avramova L., Cooks R. G., Thompson D. H. (2020). ACS Comb. Sci..

[cit30] Taylor C. J., Felton K. C., Wigh D., Jeraal M. I., Grainger R., Chessari G., Johnson C. N., Lapkin A. A. (2023). ACS Cent. Sci..

[cit31] Hase F., Roch L. M., Kreisbeck C., Aspuru-Guzik A. (2018). ACS Cent. Sci..

[cit32] Schweidtmann A. M., Clayton A. D., Holmes N., Bradford E., Bourne R. A., Lapkin A. A. (2018). Chem. Eng. J..

[cit33] Wang Y., Chen T.-Y., Vlachos D. G. (2021). J. Chem. Inf. Model..

[cit34] Häse F., Aldeghi M., Hickman R. J., Roch L. M., Aspuru-Guzik A. (2021). Appl. Phys. Rev..

[cit35] Shields B. J., Stevens J., Li J., Parasram M., Damani F., Alvarado J. I. M., Janey J. M., Adams R. P., Doyle A. G. (2021). Nature.

[cit36] Torres J. A. G., Lau S. H., Anchuri P., Stevens J. M., Tabora J. E., Li J., Borovika A., Adams R. P., Doyle A. G. (2022). J. Am. Chem. Soc..

[cit37] Hwang Y.-J., Coley C. W., Abolhasani M., Marzinzik A. L., Koch G., Spanka C., Lehmann H., Jensen K. F. (2017). Chem. Commun..

[cit38] Coley C. W., Thomas III D. A., Lummiss J. A., Jaworski J. N., Breen C. P., Schultz V., Hart T., Fishman J. S., Rogers L., Gao H. (2019). et al.. Science.

[cit39] Christensen M., Yunker L. P., Shiri P., Zepel T., Prieto P. L., Grunert S., Bork F., Hein J. E. (2021). Chem. Sci..

[cit40] Gromski P. S., Henson A. B., Granda J. M., Cronin L. (2019). Nat. Rev. Chem..

[cit41] Shen Y., Borowski J. E., Hardy M. A., Sarpong R., Doyle A. G., Cernak T. (2021). Nat. Rev. Methods Primers.

[cit42] Churski K., Korczyk P., Garstecki P. (2010). Lab Chip.

[cit43] Cook A., Clément R., Newman S. G. (2021). Nat. Protoc..

[cit44] Weiberth F. J., Powers M. R., Gallin C., McDonald D. (2018). Org. Process Res. Dev..

[cit45] Rohrbach S., Šiaučiulis M., Chisholm G., Pirvan P.-A., Saleeb M., Mehr S. H. M., Trushina E., Leonov A. I., Keenan G., Khan A. (2022). et al.. Science.

[cit46] Hsieh H.-W., Coley C. W., Baumgartner L. M., Jensen K. F., Robinson R. I. (2018). Org. Process Res. Dev..

[cit47] Whitesides G. M. (2006). nature.

[cit48] Jensen K. F. (2017). AIChE J..

[cit49] Deck L.-T. , Eyke N., Hart T. and Jensen K. F., Electromechanically driven oscillatory flow in fluidic systems, US Pat., U.S. Patent Application WO2021222334A1, 2021

[cit50] Graham R. L. (1969). SIAM J. Appl. Math..

[cit51] Della Croce F., Scatamacchia R. (2020). J. Sched..

[cit52] Ashworth I. W., Frodsham L., Moore P., Ronson T. O. (2021). J. Org. Chem..

[cit53] Kandasamy K., Vysyaraju K. R., Neiswanger W., Paria B., Collins C. R., Schneider J., Poczos B., Xing E. P. (2020). J. Mach. Learn. Res..

[cit54] Frazier P. I. , arXiv, 2018, preprint, arXiv:1807.02811, 10.48550/arXiv.1807.02811

[cit55] Kushner H. J. (1964). J. Basic Eng..

[cit56] Naito Y., Kondo M., Nakamura Y., Shida N., Ishikawa K., Washio T., Takizawa S., Atobe M. (2022). Chem. Commun..

[cit57] Haas C. P., Lubbesmeyer M., Jin E. H., McDonald M. A., Koscher B. A., Guimond N., Di Rocco L., Kayser H., Leweke S., Niedenfuhr S. (2022). et al.. ACS Cent. Sci..

[cit58] Deem M. C., Hein J. E. (2023). J. Org. Chem..

[cit59] Bradford E., Schweidtmann A. M., Lapkin A. (2018). J. Global Optim..

[cit60] Felton K. C., Rittig J. G., Lapkin A. A. (2021). Chem.: Methods.

