# Supplementary material for: Parallel multi-droplet platform for reaction kinetics and optimization
Source: Chem Sci. 2023 Aug 4;14(33):8798–809. doi: 10.1039/d3sc02082g (PMC10445457; doi:10.1039/d3sc02082g)
Supplement: SC-014-D3SC02082G-s001 [file SC-014-D3SC02082G-s001.pdf]

Electronic Supplementary Material (ESI) for Chemical Science.
This journal is © The Royal Society of Chemistry 2023

Supplementary Information
Parallel multi-droplet platform for reaction kinetics and optimization

Natalie S. Eyke,[a] Timo N. Schneider,[a] Brooke Jin,[a], Travis Hart[a], Sebastien Monfette[b], Joel M. Hawkins[b], Peter D. Morse[b], Roger M. Howard[b], David M. Pfisterer[b], Kakasaheb Y. Nandiwale[b], and Klavs F. Jensen[a*]

[a] *Department of Chemical Engineering, Massachusetts Institute of Technology, Cambridge, MA 02139, USA*

[b] *Pfizer Worldwide Research and Development, 445 Eastern Point Rd, Groton, CT 06340, USA*

**email kfjensen@mit.edu*

Contents

Page No.

S1 Platform construction details **1**

S2 Development of the photochemistry module **2**

S3 Scheduling **3**

S4 Droplet evaporation **13**

S5 Bayesian reaction optimization with Dragonfly **17**

S1 Platform construction details

A schematic of the platform is provided in Figure 1, photos of the constructed platform are included in Figure S1, a 3D rendering of the reactor tower design is shown in Figure S2, and a sketch of a sample layout of all ten reactor towers and all other system hardware is shown in Figure S3. The platform includes a liquid handler (Gilson GX-271), three syringe pumps (one to assist with the liquid handling operation, one to convey droplets to and from the reactors, and one to inject droplets of rinse solvent into the platform; all are Harvard PHD ULTRA infuse/withdraw syringe pumps, Harvard Apparatus part number 70-3007), ten-channel selector valves upstream and downstream of the reactor bank, ten reactor "towers" that incorporate all of the hardware necessary to support each reactor (SI subsection S1.1), an internal injection valve (which enables HPLC injection volumes of as little as 20 nanoliters, thus eliminating the need to dilute reacted droplets to avoid saturating the detector; VICI Valco C84H-1574-.02EUHA), and an HPLC that is remotely triggered by the control software once the droplet reaches the internal injection valve. All of the hardware is connected according to the schematic in Figure 1 using 0.02" ID, 1/16" OD FEP tubing.

Materials of construction and system flow rates were selected to minimize trailing of rinse droplets that are pumped through the platform between each reaction, which would otherwise dilute subsequent droplets and hinder reproducibility. To satisfy our operating limits of 200°C and 300psi and to allow for broad chemical compatibility, we selected compatible materials of construction for all components, and we had customized six-port, two-position valves to control each reactor channel constructed by VICI Valco to meet both our pressure limit and chemical compatibility requirements.

S1.1 Reactor towers

A "tower" was designed to house all of the accompanying hardware for each reactor channel. Each tower includes a mount for the reactor as well as the backboard that dissipates heat from the Peltier cooler behind the reactor by the attached heat sink and fan, a mount for the reactor valve above the reactor, and a control board slot that is designed to protect the control board from contact with liquid. The LED board is not pictured in the figure of the reactor tower design (Figure S2) ; in practice, it is stored across from the reactor, at the front-right corner of the tower depicted in Figure S2.

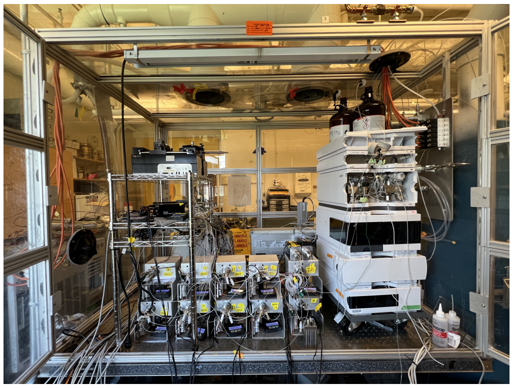

(a) Front view of the platform: reactor bank, pumps, and HPLC.

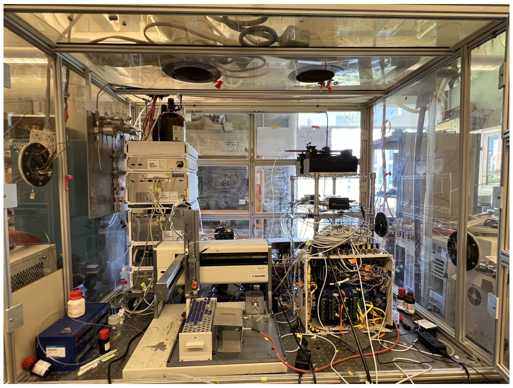

(b) Rear view of the platform: liquid handler and control box.

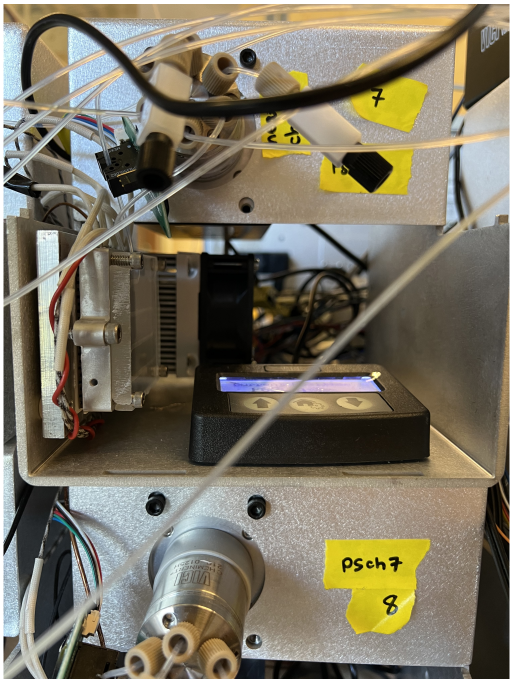

(c) Front view of reactor towers showing the reactor, valve, ancillary tubing, heat sink, fan, and valve controller.

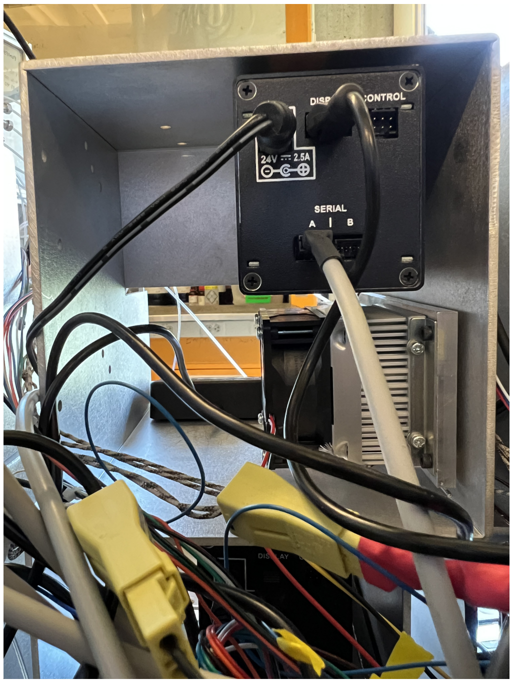

(d) Rear view of reactor towers showing the controllers and power supplies for the cartridge heaters and valve.

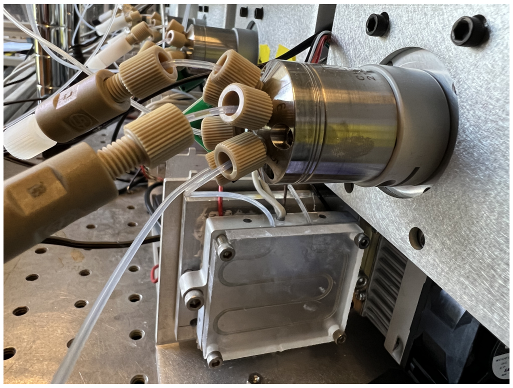

(e) Side view of the bank of reactor towers showing the valve head and reactor.

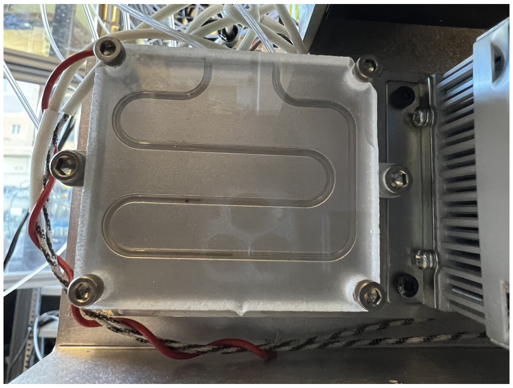

(f) Close-up of the reactor showing the flowpath and cables for temperature control.

Fig. S1 Images of the constructed platform.

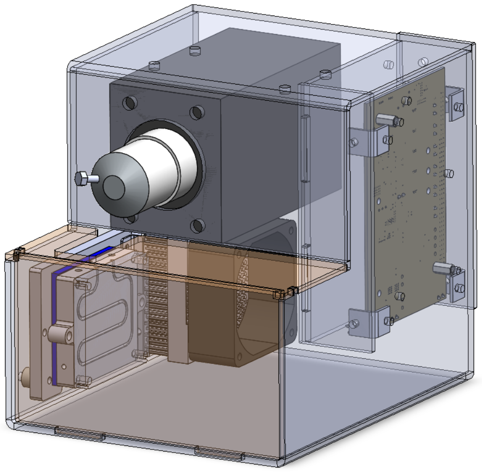

Fig. S2 SolidWorks sketch of finalized reactor tower (LED board and related hardware absent).

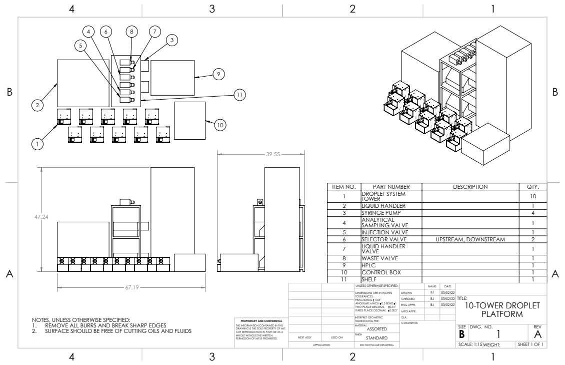

Fig. S3 SolidWorks sketch of full platform assembly. The sketch shows the reactor towers offset from one another to ensure that adjacent towers don't impact each others' cooling effectiveness.

Reactor temperature control is achieved through the use of Watlow cartridge heaters, Watlow control modules (for which corresponding software exists that can be integrated with LabVIEW), and Peltier coolers. For more information about the Peltier coolers, see Subsection S2. To accommodate the heaters, ports are drilled in the reactor behind the reactor tubing. A thermocouple is positioned within the reactor for temperature monitoring and control.

S1.2 Circuit board

A customized circuit board is used to control the Peltier cooler, the LED board, and two fans (one that cools the LED board and one that cools the heat sink attached to the Peltier cooler). The board is connected to two AC-to-DC switching power supplies, one that produces a 12V supply for the fans and Peltier (Mean Well part number SE-600-12) and one that produces a 15V supply for the LED board (Mean Well part number LRS-350-15). Control is managed by an ATmega4809 microcontroller (Microchip Technology Inc.) installed on the board. To enable the microcontroller to communicate via USB, we started by uploading the jtag2updi sketch (https://github.com/ElTangas/jtag2updi) to an Arduino Uno connected to the computer via USB, which converts the Uno into a UPDI programmer for the ATmega4809. Then, we soldered connections between specific pins on the microcontroller and Uno, which allowed us to use the Arduino IDE to burn a bootloader onto the ATmega4809 via the Arduino Uno. Once these steps are completed, the microcontroller on the control board can communicate with the computer directly via USB-C. After burning a bootloader onto the microcontroller, we defined its pins and desired functionality in an Arduino® sketch and uploaded it directly to the microcontroller via USB-C. From LABVIEW, a Python node is used to establish serial communication with the board and write serial commands to it, using the commands defined in the Arduino sketch. The fans are controlled via digital pins, offering simple on/off functionality. The LED board and Peltier cooler are controlled using pulse width modulation (PWM) pins, which enable us to control the average voltage supplied to the devices between 0 and 100% in 0.39% increments: $(1/256) * 100\% = 0.39\%$, where integer values in the range [0,255] inclusive are the analog values that can be written to the pin, since it uses an 8-bit signal, and $2^8 = 256$.

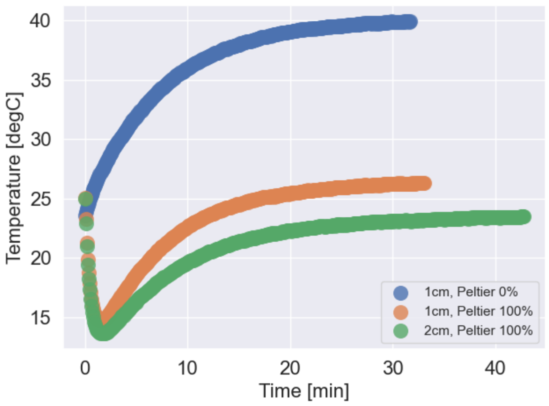

Fig. S4 Reactor temperature response to LEDs at 100% power, for various reactor-LED board separation distances and Peltier power levels.

S2 Development of the photochemistry module

The first photochemistry module development objective that we addressed was to identify the minimum distance between the LED board and the reactor that allowed for temperature control below 25°C when the LEDs and Peltiercooler are operated at 100% power. We found that a separation distance of 2cm resulted in satisfactory temperature control (Figure S4). We started by examining the temperature response when the Peltier isn't activated and the LED board is located at the minimum reasonable separation distance of about 1cm. As Figure S4 shows, under these conditions, the reactor temperature reaches 40°C, which highlights the need for a Peltier cooler and an investigation of its heat transfer capabilities. At the same separation distance of 1cm, when the Peltier is operated at full power, the temperature equilibrates slightly above room temperature, at around 26°C. When the separation distance is increased to 2cm, the temperature equilibrates around 24°C, which satisfies our temperature control specification.

In Figure S4, the temperature traces corresponding to the two full-power Peltier experiments show an initial rapid dip in temperature, followed by a more gradual rise in temperature that continues until a steady-state is reached. This behavior is a result of the fact that we did not explicitly seek to control the temperature in these experiments, but rather to monitor the temperature response when the Peltier was operating at its maximum capacity. The efficiency of the Peltier declines with time as its heat sink temperature rises; eventually, the temperature gradient in the heat sink reaches a steady-state, and therefore so does the efficiency of the Peltier and the temperature of the reactor. When reactions are performed on the platform, the temperature is explicitly controlled at a set value to prevent temperature swings like those depicted in Figure S4.

S3 Scheduling

When developing the scheduling aspect of the control software, in addition to minimizing total run time, we also sought to maximize utilization, or what fraction of the reactors are in-use in a given experiment (Figure S5). We used our analysis of utilization to determine how many parallel reactors to include in the platform. In our platform, each reaction is bookended by bottleneck operations: the liquid handler upstream, and the analysis downstream. Other hardware operations (droplet transport and rinsing) are generally much quicker than the liquid handler and analytical method and therefore they are unlikely to create bottlenecks. Peak utilization, or the maximum utilization observed in a particular experiment, is directly related to the ratio of a characteristic residence time used in the batch of reactions to either the liquid handler preparation time or the length of the analytical method, whichever is longer. The exact relation for full utilization is:

$$n_{\text{reactors}} \leq \tau_{\text{reaction}} / \tau_{\text{bottleneck}} \tag{S1}$$

As an example, consider a scenario in which the analytical method is very short (say, two minutes) and it takes the liquid handler three minutes to prepare a reaction droplet (such that the liquid handler is controlling): in this case, reaction residence times would need to be on the order of thirty minutes in order for all ten reactors to be needed to run the experiments. Reducing the reactor quantity increases the average utilization, but also radically increases the processing time in cases where residence times are longer. Since we consider thirty minutes to be a reasonably representative reaction residence time, the utilization assessment supported our decision to have ten reactors operating in parallel. The utilization assessment also highlights the value of minimizing the length of the bottleneck operations (which we considered while optimizing the liquid handler droplet preparation procedure, see Subsection S3.2).

The output of the scheduling algorithm is passed to a LABVIEW virtual instrument (VI) that orchestrates all of the hardware necessary to perform the parallel reactions. At the outset of an experiment, the software begins by performing all hardware- and software-related initializations, and then the software starts a timer that governs all subsequent operations. Once the timer starts, each of the seven

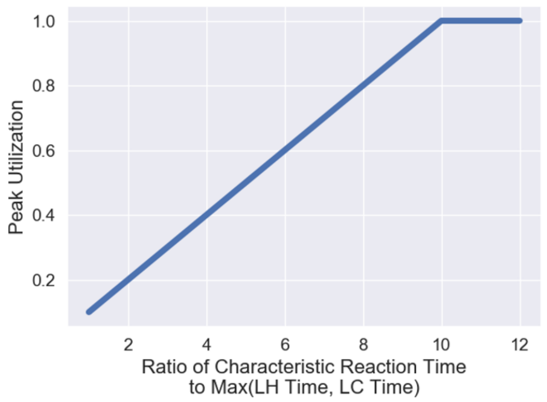

Fig. S5 Relationship between relative operation times and peak utilization for the case of ten reactors.

discrete hardware operations (liquid handler, transport, etc.) independently oscillates between an "idle" and a "running" state, according to the schedule and timer. Some of the hardware operations (droplet transport from injection valve to reactor, the reactions themselves, and analysis) require both the schedule and positive detection of the droplet at a particular phase sensor in order to transition from their idle to their running state. The droplet transport sequences manage all necessary valve openings and closures. The reactor control sequences exclusively manage temperature: each reactor accesses its own temperature schedule and starts navigating to its next temperature when its current reaction finishes (with buffer to allow the current droplet enough time to exit the reactor), which helps ensure that the reactor reaches the next temperature before the next reaction starts.

S3.1 Additional scheduler visualization

For the purposes of visualization, a simplified version of the scheduling algorithm was constructed in which all operations that occupy the main flowpath (transit to/from the reactor as well as both rinse/vent operations) were combined into one operation. Visuals of a series of scheduling cases of varying complexity are provided in Figure S6. The images verify that the algorithm moves each droplet through the platform in the expected ways (from the liquid handler, to the main flowpath, to the assigned reactor, and then back to the main flowpath, and finally on to analysis), and that there are no collisions, as desired.

The scheduling algorithm requires the following input from the user: the length (in seconds) of each hardware operation (including droplet preparation, transport to/from the reactor, both rinse/vent operations, and the analytical method), along with the residence time of each reaction, and a list of which reactors are operational. The exact length of droplet preparation will vary depending on the number of stock solutions being combined and the location of the stock solutions on the liquid handler well plate; the user must specify the longest potential operation time to avoid faults.

S3.2 Optimizing liquid handler-based droplet preparation for speed and accuracy

Prior experimentation exposed large discrepancies between the target droplet concentrations specified in the control software and the actual concentrations detected in prepared droplets. As an illustration of the magnitude of the problem, the average absolute error in in the starting material concentration across an early set of validation reactions was 88%, an order of magnitude greater than what we would consider acceptable. Charge error can arise due to suboptimal design and operation of the liquid handler that is used to prepare the reaction droplets.

To address this problem, we took a closer look at the liquid handler operation. Every liquid handling variable can be customized to suit varying accuracy and speed requirements, from the size of the syringe used to prepare droplets, to the identity of the fluid used as a buffer between the transfer fluid and the reaction droplet, to the number of times the droplet is oscillated inside the liquid handler needle to promote homogenization. We thoroughly investigated the influence of these parameters on the speed and accuracy of the droplet preparation procedure.

Droplet preparation begins once the platform control software finishes calculating the volume of each stock solution that the liquid handler must collect in order for the final droplet to contain the user-specified concentrations of all the desired components. The liquid handler then withdraws the requisite volumes of each stock solution one after another into its needle (see Figure S7), “stirs” the stacked sub-droplets through repeated pump infuse-withdraw operations until uniformity is achieved, and then transports the homogenized droplet into an injection valve that is connected to the reactor.

The experiments described herein used a Gilson GX-271 liquid handler outfitted with a 221-by-1.5-by-1.1mm (length-by-OD-by-ID) constricted flat tip needle with a 0.45mm ID at the tip of the constriction (Gilson part number 27067373) and a rack with wells for 96 12 x 32 mm vials (2 mL) (Gilson part number 2504609). A Harvard PHD ULTRA infuse/withdraw syringe pump (Harvard

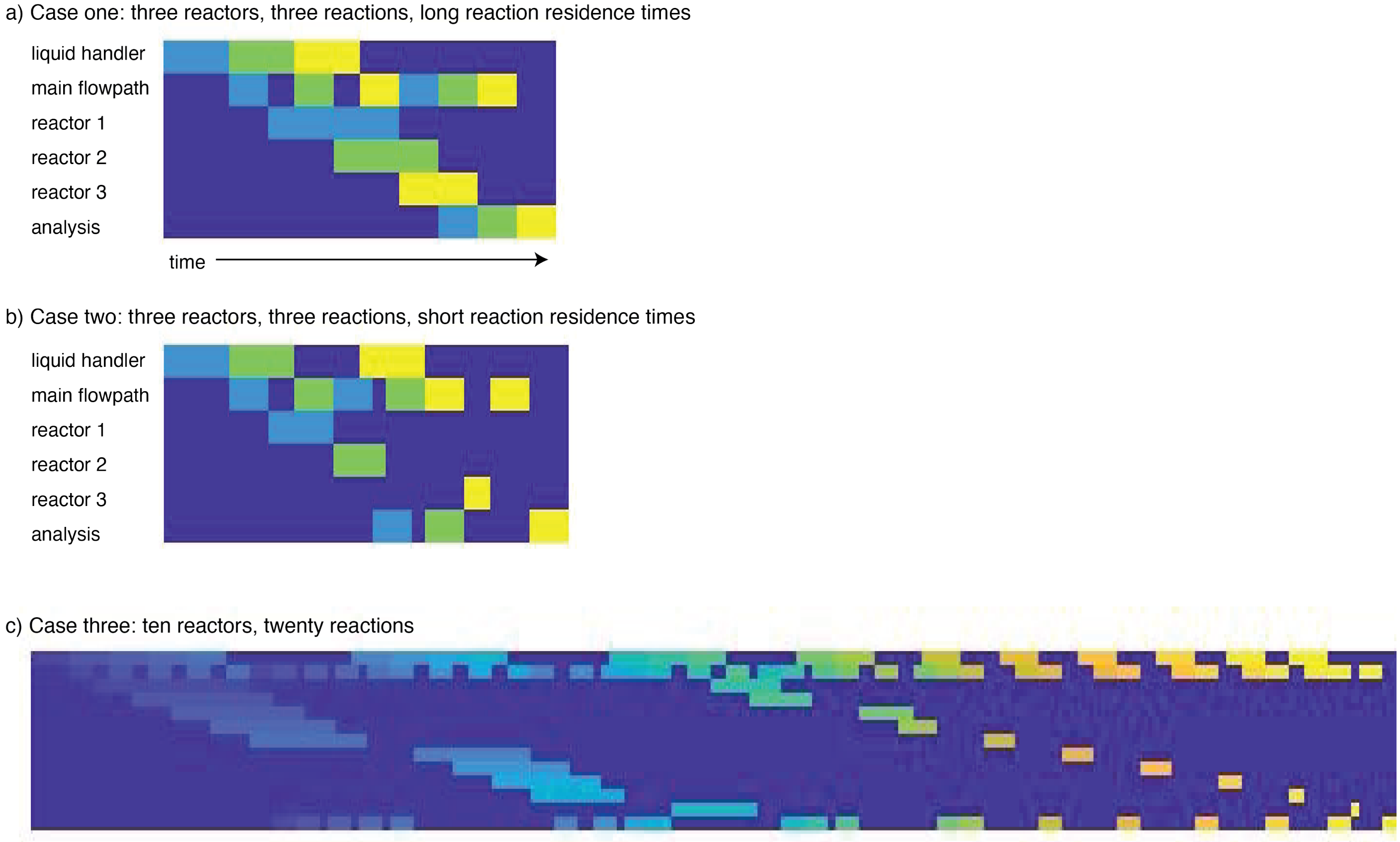

Fig. S6 Visualization of simplified scheduling algorithm in which all operations that occupy the main flowpath were combined. Each unique reaction is represented using a distinct color. Dark blue represents background or unused time. (a) and (b) are both cases in which three reactions being assigned to three parallel reactors, and provide confirmation of the sketch in Figure 4: when the reactions are long compared to the other operations, droplet preparation can be back-to-back (case depicted in (a)); but when reactions are relatively short, as in (b), the algorithm imposes a pause between the preparation of the second and third droplets. (c) shows the algorithm handling a much more complex case, in which twenty reactions are assigned to ten parallel reactors.

Apparatus part number 70-3007) was used to aspirate the stock solutions, stir the droplet, and transport the droplet to the injection valve. Several different syringes with varying diameters were used throughout development. Standard HPLC vials (Agilent part number 5182-0715) were used to store stock solutions in the liquid handler rack in all experiments. FEP tubing with ID 0.02", OD 1/16" was used throughout: to connect the syringe pump to the head of the liquid handler needle and to transport the droplet throughout the platform. Droplet transport on the platform involved navigating through constrictions created by 1) the injection valve; 2) an 11-port, 10-position selector valve (VICI Valco, C5H-3720EUHAY); 3) a T-junction (Upchurch Scientific); and 4) a 6-port, 2-position reactor valve (VICI Valco, C2-3726EUHAY). Control software for all equipment was written using a combination of MATLAB®† and LABVIEW®‡.

Liquid handling optimization: methods

To improve the accuracy and reproducibility with which the liquid handler creates droplets, we began by listing all of the hardware and operational parameters that have the potential to influence the accuracy of the droplet preparation process. Throughout our analysis, we were also mindful of the time necessary to complete one droplet preparation cycle, because the native implementation of the liquid handler involves preparing just one droplet at a time, so the droplet preparation step is a bottleneck in the parallelized system (see Subsection S3). Table S1 shows all of the influential parameters that were identified, along with an indication of which performance factors the parameter has the potential to influence, and a note about how much control we have over the parameter.

To study the influence of these parameters on the accuracy and speed of droplet preparation, we programmed the liquid handler to prepare a series of droplets while we varied the parameter settings and the hardware. The exact experimental procedure evolved over time. For the first round of experiments, we analyzed one-component droplets containing biphenyl (Sigma-Aldrich, $\geq$ 99.9%)

† https://www.mathworks.com/products/matlab.html
‡ https://www.ni.com/en-us/shop/software/products/labview.html

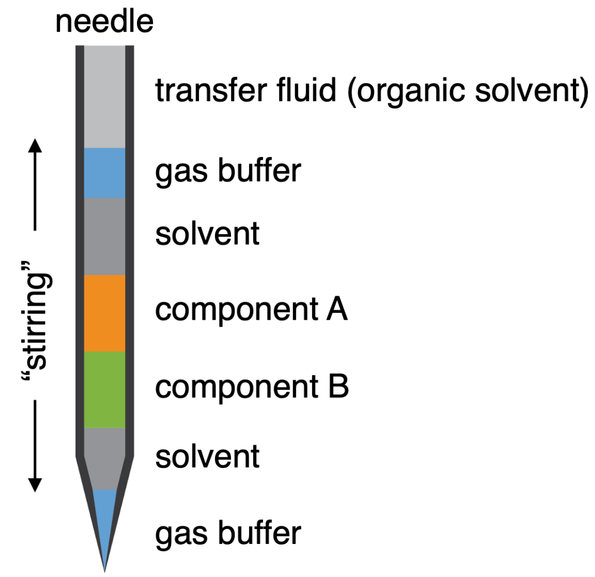

Fig. S7 Depiction of a prepared two-component droplet, between the stock solution aspiration step and the stirring step

in acetonitrile (Sigma-Aldrich, gradient grade, $\geq$ 99.9%). At the outset of experimentation, a calibration curve for biphenyl was constructed. Stock solutions for all subsequent experimentation were designed to be within the linear detector response range verified during calibration. To test droplet preparation parameters, we used the Gilson liquid handler to prepare droplets of biphenyl in acetonitrile at various concentrations, and injected these through the Gilson injection valve and into the sample loop. Once the Gilson injection valve switched, we used the carrier syringe pump (Harvard PHD ULTRA infuse-only, Harvard Apparatus part number 70-3005) to infuse the droplet out of the sample loop and into a 2-mL HPLC vial by disconnecting the downstream sample loop fitting and positioning it in the insert. It was necessary to repeat each set of conditions three times in order for the vial to contain enough material for analysis. Vials were stored in the refrigerator in between repeat experiments to minimize evaporation of the tiny volumes of sample. Once a given set of conditions had been repeated three times, we analyzed the sample by HPLC, and used the calibration curve to compute the concentration of biphenyl in the droplet and compared that value to the target value that we'd specified in the control software.

To accelerate parameter screening, we later augmented the experimental procedure by reprogramming the liquid handler to inject prepared droplets directly into HPLC vials. We installed 200-μL conical inserts into each of the collection vials to eliminate the need to repeat experiments multiple times in order to collect enough material for analysis. We continued to repeat each set of conditions three times, but analyzed each run separately to monitor variance on a condition-by-condition basis. With this approach, we studied both one-component and two-component droplets, using biphenyl as the first component, as before, and 4'-bromoacetophenone (Sigma-Aldrich, 98%) as the second component. At the outset of experimentation, a calibration curve for 4'bromoacetophenone was constructed. Stock solutions for all subsequent experimentation were designed to be within the linear detector response range verified during calibration. Multicomponent droplets are more challenging to prepare accurately than single-component droplets: the minimum aspiration volumes decrease and the number of necessary rinse vial dips increases; each vial dip represents an opportunity for material that is already in the needle to diffuse out of it. Within the droplet itself, the diffusion length scale increases. For a portion of these experiments, we isolated the non-stirring-related parameters from the stirring-related ones by removing the stirring operation from the preparation procedure and mixing droplets manually instead. In these cases, we mixed the droplets manually after preparation was complete by repeatedly inverting the vial. Development in this phase focused on exploring the influence of the syringe volume (100, 250, and 500μL syringes), the droplet preparation volume (25 to 120μL), the withdrawal flow rate used during stock solution aspiration (100 to 500μL/min), all stirring-related parameters (total stir volume [25 to 250μL], quantity of stirring operations [up to 10], stirring flow rate [100 to 500μL/min]), the volume and makeup of the buffer between the droplet and the transfer fluid, and the identity of the transfer fluid.

Later, it became clear that hardware downstream of the liquid handler itself also had an effect on the composition of the droplet. Thus, a third version of the experimental protocol was necessary, in which droplets were prepared as before, and then droplets were injected onto the system, infused through the system with the help of the carrier syringe pump and pressurized carrier gas, and finally collected in a vial with 200-μL conical insert stored in a pressure bomb at the system outlet. Injection onto the system was achieved via two different mechanisms. The first injection mechanism is the original approach, which involves injecting the droplet through the liquid handler needle, through the needle injection port on the Gilson injection valve, and into a sample loop installed on the valve. Problems with hang-up in the Gilson injection valve motivated us to also test a "withdrawal" approach: we installed a six-port, two-position valve (VICI Valco C2-3726EUHAY) with a sample loop constructed out of 7.5" of 1/16" OD, 0.02" ID FEP tubing on the

Parameter	Effect on accuracy?	Effect on speed?	Controllability
Syringe volume	Y	N	Syringes are available in varying volume increments from 100μL to 8mL
Transfer fluid properties	Y	N	Very controllable - fluids with a wide array of properties are available
Target preparation volume	Y	Y	Controllable in the range [0,500]μL
Aspiration flow rate	Y	Y	Controllable in the range [0,13]mL/min
Buffer volume	Y	Y	Controllable in the range [0,500]μL
Buffer fluid properties	Y	N	Very controllable - fluids with a wide array of properties are available
Number of stirs	Y	Y	Infinitely controllable
Stir flow rate	Y	Y	Controllable from 0 up to the syringe pump pressure limit (varies with syringe size)
Stir volume	Y	Y	Controllable in the range [0,500]μL
Needle geometry	Y	N	Not very controllable - a number of different probes are available from Gilson, but delivery timelines are long, and the needle-swapping procedure itself can damage the hardware in a way that negatively affects accuracy
Ratio of target droplet concentration to stock solution concentration	Y	N	Controllable in the range [0,1]
Mixing chamber above needle	Y	N	Any length of tubing with outer diameter $\leq$ 1/8" can be easily installed; through custom machining, a wide range of inner diameters, geometries, and lengths are possible

Table S1 Impact and controllability of liquid handler hardware and operational parameters

transfer line between the liquid handler syringe pump and the liquid handler needle, and withdrew the droplet through the top of the liquid handler needle, into the transfer line and into the sample loop. After each experiment performed using this protocol, the tubing and hardware connecting the injection valve to the system outlet had to be rinsed; for this purpose, a train of three 50-μL droplets of the solvent being used for the experiment was infused from a point upstream of the injection valve to the tubing terminus in the pressure bomb using another Harvard Apparatus infuse-only syringe pump. During this phase of development, we separately also tested the influence of installing a "mixing chamber" (piece of large-ID [1/16"] FEP tubing) above the liquid handler needle, and stirring the droplet there.

One of the key performance-influencing aspects of the preparation procedure is the need for a buffer of some kind between the droplet and the transfer fluid to prevent the droplet's contents from diffusing into the transfer fluid. In prior implementations of the automated droplet reactor, a gas buffer was used to separate the droplet from the transfer fluid; experiments indicated that the presence of gas anywhere in the transfer line had a negative effect on the accuracy of the preparation process, so alternatives to the gas buffer were explored, including a series of perfluorinated oils exhibiting various properties. Since they're immiscible with most common organic solvents, perfluorinated oils are frequently used in a wide variety of segmented flow applications (e.g.[61–65]). A selection of the oils we tested is listed in Table S2 along with relevant physical properties. We included tetrahydrofuran in the table as well as a reference because it's a solvent we commonly use as the transfer fluid. Ideally, in addition to the properties listed in the table, we would also know each oil's wetting behavior on the material of the liquid handler needle, along with that of each solvent; this would help us predict the phase intermixing behavior in the vicinity of the oil-drop interface. To prevent droplet-oil intermixing while also preventing the oil from dripping out of the tip of the needle, we want an oil with relatively high viscosity, relatively low density, and relatively high surface tension. The oil should also have low compressibility (to maximize alignment between the displacement volume of the syringe plunger and the volumes aspirated from stock solutions) and low affinity for the reactant and reagent compounds.

We also conducted experiments to directly examine the performance of the liquid handler syringe pump, which operates by turning a threaded screw with a backplate mounted onto it. The plunger of the syringe is secured to the backplate. The accuracy therefore depends on the pitch of the screw thread, the diameter of the syringe, and the temporal tolerance of the pump's on/off commands. To improve accuracy, we found it necessary to install washers on the plungers themselves to eliminate gaps between the backplate mounting mechanism and the plunger base. We programmed the syringe pump to withdraw volumes of magnitudes similar to those used during droplet preparation (5 to 25μL), and measured the resulting change in the liquid front at various points in the transfer line. Then, using the known internal diameter of the tubing, the actual change in volume could be determined. It wasn't possible to

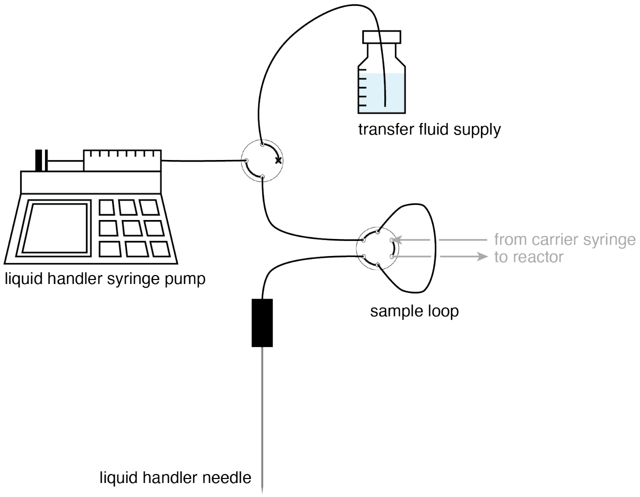

Fig. S8 Diagram of hardware arrangement allowing the droplet to be withdrawn into a sample loop, rather than injected through a needle seat

Fluid	Viscosity [cP]	Density [g/mL]	Surface tension [mN/m]
Perfluorohexanes	0.67	1.68	11[b]
Perfluorodecalin	5.1	1.93	19[c]
FC-70[a]	24	1.94	18
Tetrahydrofuran	0.48	0.89	26

Table S2 Properties of selected perfluorinated oils. [a]Properties of FC-70 taken from the data sheet provided by the manufacturer: https://multimedia.3m.com/mws/media/64891O/fluorinert-electronic-liquid-fc-70.pdf. [b]See[66]. [c]See[67]. Tetrahydrofuran included as reference.

make this assessment in the needle, which is opaque; therefore assessments were confined to the transfer line upstream of the needle: specifically, upstream of the injection valve; downstream of the injection valve, between the valve and the mixing chamber; and in the mixing chamber.

The preparation volume has competing effects: larger preparation volumes translate to larger aspiration volumes, which improves accuracy because whatever absolute error exists in the pump itself becomes a smaller fraction of the total. However, larger droplets are also more difficult to homogenize. Thus, there are strong interactions between the setting for the preparation volume and the settings that influence the quality of stirring. A related factor is the relative values of the target concentration for the droplet and the concentration of the stock solution: smaller target/stock ratios translate to smaller aspiration volumes.

The droplet is stirred via repeated infuse/withdraw operations of the pump, which causes the droplet to oscillate inside the liquid handler needle. Three distinct parameters can be used to control the extent of droplet mixing: the number of stirring operations that are performed, the volume displaced per stroke, and the syringe pump flow rate applied during stirring. The quantity of stirring operations has to be high enough to homogenize the droplet. However, each discrete stirring operation is costly in terms of preparation time; therefore, the quantity of stirring operations also has to be low enough to avoid converting the droplet preparation step into a significant bottleneck. The displacement volume should be large enough to allow for full recirculation within the droplet. The flow rate during stirring should be high enough to mix the droplet effectively without imparting so much energy to the fluid that the interface between the droplet and the oil buffer is disrupted.

Stirring is critical in this application because the stacking of the stock solution sub-droplets (see Figure S7) in the needle during preparation implies that the diffusion length scale is equal to the half-length of the droplet, which, with reference to the needle internal diameter of 1.1mm, is about 25mm for a 50μL droplet. Assuming a diffusion coefficient D of 1×10^{-9} m^2/s, this translates to a diffusion time scale $\tau_D \sim$ (droplet half-length)$^2/D$ that is on the order of days. The oscillatory stirring motion imposed on the droplet by the syringe pump, combined with the resulting shear at the needle wall, creates recirculating vortices inside the droplet, thus reducing the diffusion length scale from the droplet half-length to the needle radius.

Liquid handling optimization: results and discussion

The first round of liquid handler droplet preparation optimization involved collecting droplets injected through the injection valve sample loop. At this stage of experimentation, three droplets had to be prepared sequentially under fixed conditions and collected into the same vial in order to collect enough material for analysis; therefore the results at this stage represent an averaging of three runs. Under these conditions, apparent variation is reduced, and nondirectional charge variation (i.e. that which is centered around a mean charge error of zero) becomes harder to detect due to the central limit theorem. Further, since each preparation cycle takes a few

minutes, there is more time for diffusion to homogenize the overall sample, which can hide the effects of incomplete stirring.

We compared charge error produced by an 8-mL syringe (Harvard Apparatus part number 70-2267, 9.525mm diameter) and that produced by a 2.5-mL syringe (Harvard Apparatus part number 70-2269, 4.75mm diameter). Under otherwise identical conditions, switching to the smaller syringe reduced absolute charge error from 13% (std. dev. 9%, N=3, with each individual run representing an average of three distinct droplets) to 3% (std. dev. 2%, N=3). This is consistent with our expectations based on the mechanism of operation of the syringe pump. As described in Subsection **??**, syringes with smaller diameters offer finer volume control, because a given rotational translation of the thread that drives the plunger corresponds to a smaller absolute volume for the syringe with the smaller diameter than for the syringe with the larger diameter. In the next phase of development, this effect was explored further using even smaller syringes.

Experiments at this phase demonstrated the crucial role played by the stirring operation. Consider Figure S9: when a droplet hasn't been completely mixed before entering the sample loop, the portion of the droplet captured by the sample loop will contain relatively high concentrations of some components and relatively low concentrations of others, thus increasing the apparent error. The sample loop has to be overcharged, as shown in Figure S9, to prevent the droplet from breaking up when the valve switches from the atmospheric pressure of the liquid handler to the elevated pressure of the platform. Prior to stirring optimization, when entire prepared droplets were analyzed, the average absolute charge error was much lower than when only the portion of the droplet that entered the sample loop was analyzed: 3% versus 24%, respectively. This round of experiments further showed the benefit of increasing the number of stirring operations: under otherwise fixed conditions, the average absolute error decreased from 42% to 38% to 5% as the number of stirring operations increased from 2 to 4 to 8, respectively.

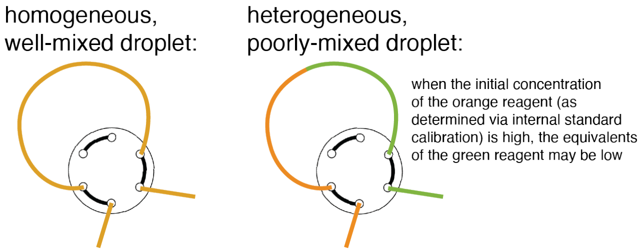

Fig. S9 Homogeneous and heterogeneous droplets captured by a sample loop

To accelerate development and improve the fidelity of measurements, we began injecting droplets directly into vials (rather than through the injection valve) and collecting droplets in 200-μL conical vial inserts, which allowed experiments to be analyzed one by one. The magnitude of the charge error observed in individual droplets is larger than when the analysis represents an averaging over three combined droplets. One drawback to this approach is that it doesn't capture the influence of droplet non-homogeneity as well as injecting through the sample loop does, because all droplets are analyzed in their entirety.

The influence of the syringe diameter and volume was explored further in an attempt to further capitalize on the trend observed when the syringe diameter decreased from 9.525mm to 4.75mm (see above) by testing a series of glass SGE syringes: 100μL (1.46mm ID), 250μL (2.3mm ID), and 500μL (3.26mm ID). However, in addition to the theoretical accuracy advantages associated with syringes with smaller diameters, experiments at this stage demonstrated that there are also subtle trade-offs associated with reducing the syringe diameter: syringes whose volumes aren't much larger than the droplet itself (such as the 100-μL syringes used in the old platform) impose very restrictive limits on the droplet preparation volume, the volume that the droplet can be displaced during stirring, and the flow rates that can be employed (due to pump-imposed pressure limits). For example, when comparing preparation with the 250μL syringe and the 100μL syringe, the restrictions on operating parameters at the 100μL volume translated to an increase in the error from 30% to 34%.

A separate operational consideration is relevant to syringe selection as well: the 2.5mL syringe from Harvard, unlike the SGE syringes, is designed for compatibility with the Harvard syringe pump. The Harvard syringe is constructed out of stainless steel and compatible with a Swagelok fitting to connect to the transfer fluid line, which ensures a good seal. The glass syringes' plungers are shorter than the Harvard ones, so without software-based monitoring of the total infused and withdrawn volume and related recovery software, the pump will occasionally pull the plunger out of the syringe entirely. For similar reasons, since the SGE syringes are made out of glass, the pump occasionally crushed them. Further, the SGE syringes aren't compatible with Swagelok fittings, so Luer-lok fittings were used instead, but these don't seal as well as Swagelok, and the large pressure drops created during withdraw operations occasionally resulted in the syringe pulling in air through the fittings. These considerations taken together supported a decision to permanently use the 2.5-mL Harvard syringe.

To definitively validate the 2.5-mL Harvard syringe's performance, we measured the displacement in the liquid front that resulted when the pump was programmed to withdraw droplet-scale volumes. The results are presented in Table S3. Most conditions were repeated multiple times to examine variability. The results show that the syringe pump outfitted with the 2.5-mL Harvard syringe is extremely accurate in the absence of any hardware in the transfer line, which further supported the decision to make it a permanent

Measurement point	Target withdrawal volume [μL]	Measured withdrawn volume [μL]
6*Between syringe and LH valve (tubing ID: 0.02")	25	26.00
	10	10.00
	10	10.00
	5	5.00
	5	5.00
	5	5.00
5*Between LH valve and mixing chamber (tubing ID: 0.02")	5	4.99
	5	5.15
	5	5.15
	5	5.15
	5	5.07
6*In the mixing chamber (tubing ID: 1/16")	25	18.07
	25	25.14
	25	25.14
	25	24.35
	25	22.00
	25	23.57

Table S3 Accuracy of 2.5-mL Harvard syringe: withdrawal volume, target versus actual

feature of the system. Downstream of the liquid handler valve, between the liquid handler valve and the mixing chamber, the accuracy decreases slightly, but the error is still tiny ($\sim 2\%$) even at the small aspiration volume of 5μL, which is representative of the smallest volumes we expect to aspirate during preparation. In the mixing chamber, accuracy decreases a lot, to nearly 8% for an aspiration volume five times greater than the minimum.

Before we made the definitive switch back to the 2.5-mL Harvard syringe, many experiments were performed using the smaller SGE syringes. For a portion of these experiments, the droplets were not stirred in order to isolate the influence of non-stirring parameters from the influence of stirring-related parameters. Stirring has an enormous effect on the outcome, and stirring parameters have significant interactions with non-stirring parameters, so the only effective way to analyze the influence of the non-stirring parameters was to mix the droplets manually post-preparation. We did this by gently and repeatedly inverting the vials.

The results of the manually-stirred droplet experiments are somewhat scattered, making them somewhat difficult to interpret, which could be due to incomplete and inconsistent mixing. The conical insert has a very narrow pore at the very bottom, where capillary action keeps fluid from flowing out when the vial is inverted, which prevents effective manual mixing. More aggressive mixing procedures, such as vortexing, weren't pursued because the droplet would have splattered, which would have increased the area of the gas-liquid interface significantly, leading to increased evaporation, which would have had its own detrimental impact on the quality of the analytical outcome.

However, the results of the manually-stirred preparation experiments are directionally informative. For example, under otherwise identical conditions, reducing the size of the gas buffer between the droplet and the transfer fluid from 40μL to 20μL reduced the average absolute error from 18% (N=3, σ=10.6) to 13% (N=3, σ=1.3). A smaller gas buffer translates to lower charge error as well as lower run-to-run variation, which can be explained by the compressibility of the gas. Compression or expansion of fluid in the transfer line naturally results in differences between the displacement of the syringe plunger and the volume that is actually aspirated from a stock solution vial.

With stirring re-incorporated into the test procedure, we built upon the observation I made about the influence of the gas buffer, and eliminated it completely by replacing the tetrahydrofuran transfer fluid with perfluorohexanes, which is immiscible with the organic solvents intended for use on the platform. Under otherwise identical conditions (except for the removal of the mixing chamber), replacing THF with perfluorohexanes reduced the error from 42% (σ=16%, N=3) to 5% (σ=2%, N=2). While the perfluorinated oil resulted in significant reductions in the measured charge error in these cases, it should be noted that the oil is not a panacea. First of all, compounds that are soluble in the perfluorinated oil will tend to diffuse into it. Second, perfluorinated oils have very high oxygen solubility (high enough that they are used as oxygen sources in a variety of applications, such as bioreactors[68], and even in medical applications where they have been shown to function as "liquid ventilators" in lung disease patients, see e.g.[69]). Many organic synthesis reactions are sensitive to oxygen. Oxygen-sensitive applications will require the perfluorinated oil to be degassed prior to experimentation.

With the perfluorohexanes transfer fluid, further experimentation focused on examining the influence of the droplet preparation volume, the withdrawal flow rate used during stock solution aspiration, and the various stirring-related parameters. Two-component droplets were used to apply greater stress to the system and better mimic realistic preparation conditions; results that follow represent

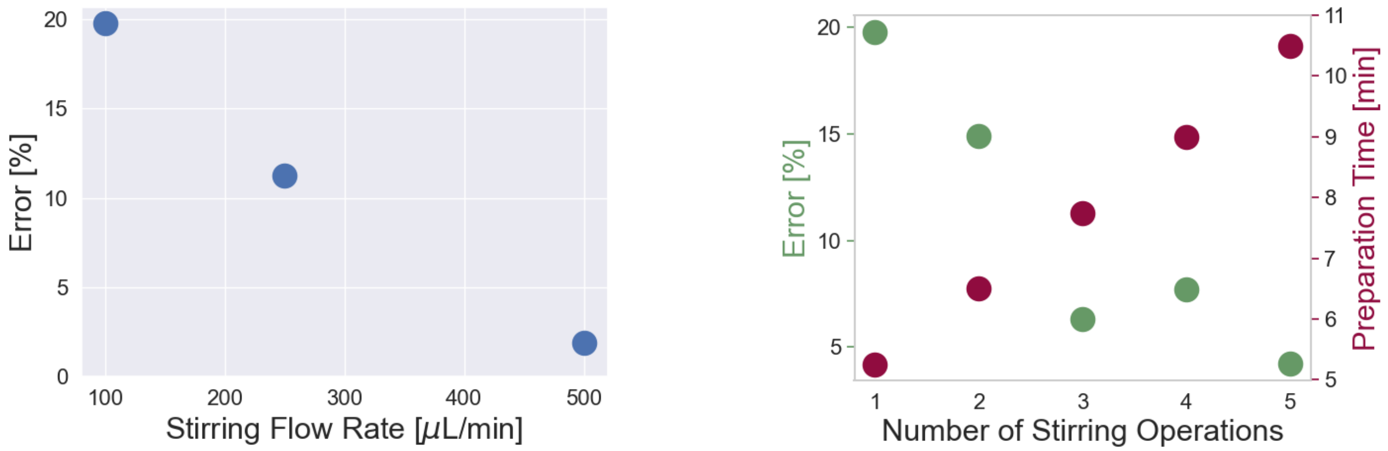

(a)

(b)

Fig. S11 Influence of stirring-related parameters on preparation error and preparation speed

the average error across the two components.

Figure S10 shows the somewhat non-intuitive result of increasing the preparation volume while holding all other parameters constant. The average error across the two components actually increases with increasing preparation volume, because a given set of stirring parameters won't homogenize large droplets as effectively as small ones.

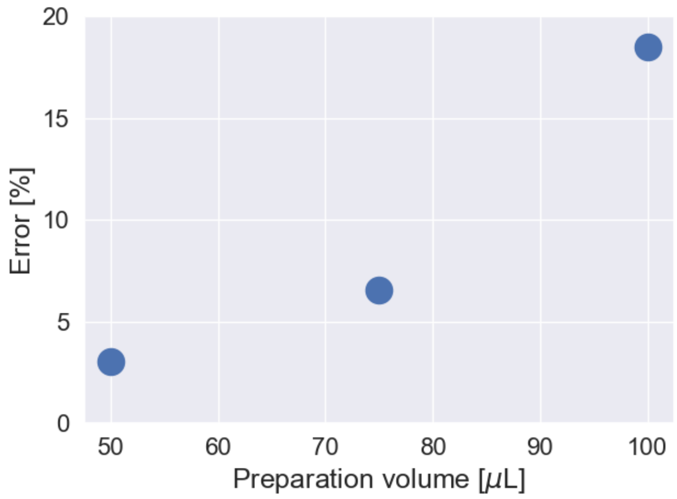

Fig. S10 Two-component average error versus preparation volume

Increasing the quality of stirring by increasing the quantity of stirring operations and the stirring flow rate results in more homogeneous droplets and lower apparent error (Figure S11). However, during droplet preparation, speed is nearly as important as accuracy, due to the potential for bottleneck formation if preparation requires too much time, and increasing the quantity of stirring operations quickly increases the total preparation time.

The flow rate used while aspirating stock solutions has a less dramatic influence on preparation time, but a significant influence on error (Figure S12). Higher flow rates during aspiration tended to result in large quantities of perfluorohexanes in the prepared droplets. The sample in Figure S12 corresponding to an aspiration flow rate of 500μL/min resulted in a droplet that was nearly 50% perfluorohexanes by volume. Interestingly, this outcome is not observed when the droplet is *prepared* using a relatively low aspiration flow rate of 100μL/min, and then *stirred* at 500μL/min; instead, the combination of slow aspiration with fast stirring results in very low error (Figure S11a).

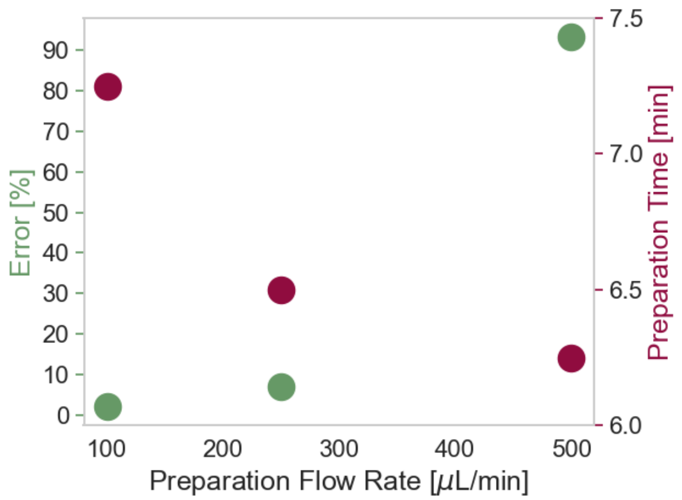

Fig. S12 Two-component average error and total preparation time versus aspiration flow rate

These analyses led us to identify operating conditions that consistently delivered droplets with discrepancies between the target and actual concentrations of less than three percent, down from the 88% average error that we observed during preliminary experimentation. Using perfluorohexanes as a transfer fluid, 50μL preparation volume, 50μL stir volume, 100μL/min preparation flow rate, 500μL/min stir flow rate, and four stirring operations, the average charge error is 2.03%. The total preparation process under these conditions takes around seven minutes, which is long enough to create a meaningful bottleneck in the system.

The optimized preparation conditions outlined above were identified by injecting prepared droplets directly into vials, without passing through the injection valve; when the injection valve was reincorporated into the workflow, perfluorohexanes was observed in the droplets. Since perfluorohexanes is the transfer fluid, the needle purge step that precedes droplet preparation was leaving perfluorohexanes hang-up in the injection valve, and the hang-up was mixing with the prepared droplets when they were injected into the valve. With reference to Figure **??**, this observation motivated me to switch from using perfluorohexanes as the transfer fluid to using it as a buffer between an appropriate rinse solvent (such as tetrahydrofuran) and the droplet.

Changing to a perfluorinated oil buffer reduced the amount of oil in the droplets that were injected into the injection valve, but didn't eliminate it; unlike when the oil was used as the transfer fluid, the oil tended to be localized at the trailing edge of the droplet. Localization of the oil near the trailing edge, where the interface between the droplet and the oil is located, suggests that the increased pressure drop needed to get the droplet through the injection valve increases the droplet's velocity, thus creating conditions that are more favorable for intermixing between the solvent and oil phases. Switching from perfluorohexanes to perfluorodecalin, which is more viscous, did not solve the problem.

To minimize the impact of this "oily edge" on the prepared droplet, we tried increasing the droplet preparation volume to 75μL, which proved to be large enough to keep the oily edge out of the sample loop with low charge error. However, the additional stirring operations necessary to homogenize the larger droplet resulted in a preparation time of eight minutes. I also tested the effect of preparing substantially larger droplets: 300μL. Ten stirring operations were necessary to achieve the same accuracy as for the smaller droplets (the stir volume was increased to 200μL to account for the larger droplet, but otherwise the optimized conditions were unaltered), but this resulted in a total preparation time of twenty minutes, which would create a major bottleneck during parallelized operation, unless the large droplet was subdivided into several discrete droplets. Each of the subdivided droplets would be of identical composition, but they could be used to study the effects of other process variables on the reaction outcome, such as temperature and residence time. For this approach to work, the droplet would need to be totally homogeneous along its entire length; to verify complete mixing, We prepared different 300μL droplets and examined different cuts of each one (Table S4). The results show that the error is low across the entire length of the droplet. At a total preparation time of around twenty minutes, a 300-μL droplet could be subdivided into six droplets of identical composition, translating to a per-droplet preparation time of under four minutes.

We used the further-optimized preparation conditions to perform an additional series of test reactions. The results of the test reactions highlighted a need for further modifications to the droplet handling procedures: all but one of the reactions had a lower starting concentration than the specified target (Figure S15), and the average signed error was enormous (compared to the optimized preparation tests) at -57%. These results imply that the droplets were being diluted, which could happen due to solvent hang-up in the injection valve and other hardware in the platform with constriction points where fluid may tend to accumulate. To examine this further, we prepared identical droplets, and passed one of them through the injection valve and sample loop only, and the other through the entire system; the resulting preparation errors were -10% and -66%, respectively, suggesting an accumulating dilution effect that got worse as the number of hardware components the droplet encountered increased.

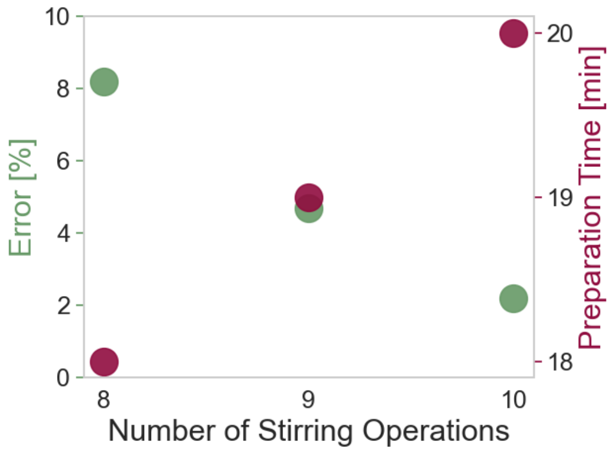

Fig. S14 Charge error and total preparation time versus quantity of stirring operations for 300uL droplets

Which cut?	Error [%]
First	0.3%
First	1.2%
Third	0.6%
Fifth	-2.6%
Sixth	1.7%

Table S4 Charge error across different cuts of different 300μL droplets

To address the problem of solvent hang-up, we switched from injecting the droplets onto the platform via the Gilson injection valve to instead withdrawing them into a VICI Valco six-port two-position valve (Figure S8). We made this change based on the hypothesis that whatever mechanism was used to seal the needle seat in the Gilson injection valve would introduce more hang-up than a simple six-port two-position valve; even if this change didn't fix the problem, it was unlikely to make it worse, since the valves are practically identical aside from the needle seat. We also added a "vent" step between reactions, in which we opened a port at the center of the platform to atmosphere while the cylinder that supplies gas to the system was also open, and let the system vent for a minute or two to dry out any solvent that might have accumulated. After the vent step was complete, we closed the open port and re-pressurized the system.

To test the effects of these modifications, we performed a series of SNAr reactions and measured the conversion as well as the charge error of the limiting reactant. The results are tabulated in Table S6. The reaction that was injected through the injection valve, rather than withdrawn into a sample loop on the VICI Valco valve, appears to have been diluted significantly. With the vent step added to an oil buffer preparation, the dilution effects are minimized.

S4 Droplet evaporation

We monitored droplet evaporation versus temperature and residence time. At temperatures above 140°C, oscillating droplets of tetrahydrofuran (THF) would evaporate within minutes, and corresponding condensate drops would form in the cooler tubing outside the reactor.

Whenever the reactor is controlled at a temperature above room temperature, oscillating droplets shrink over time due to evaporation. Unlike a reaction in a sealed, jacketed vessel, the droplet does not merely evaporate to saturate the headspace, and then cease evaporating once equilibrium is reached. Rather, several distinct phenomena contribute to a more dynamic behavior, in which volatile compounds in the droplet evaporate to saturate the headspace, and the saturated vapor condenses in the tubing outside the reactor (which is not temperature-controlled, Figure 2), thus forcing further evaporation of the droplet to maintain headspace saturation. The droplet also tends to smear along the walls as it oscillates, which increases the interfacial area between the droplet and the headspace, meaning the vapor phase is readily replenished whenever desaturation occurs.

One phenomenon that contributes to this dynamic behavior is a result of the oscillation itself. Oscillation of the fluid inside the loop pushes vapor that is saturated at the reaction temperature into the cooler tubing outside it. To the extent that the vapor cools, it will condense rather than persist in a supersaturated state. When liquid condenses outside the reactor, it tends to remain suspended there, since the organic solvents we use don't perfectly wet the FEP tubing walls. Images of condensate forming in the tubing above a reactor maintained at 60°C with an oscillating 40-μL droplet of THF are shown in Figure S16. Figure S16c shows a 6mm-long droplet of condensate that has coalesced so as to span the cross-sectional area of the tubing; the droplet's length translates to a volume of roughly

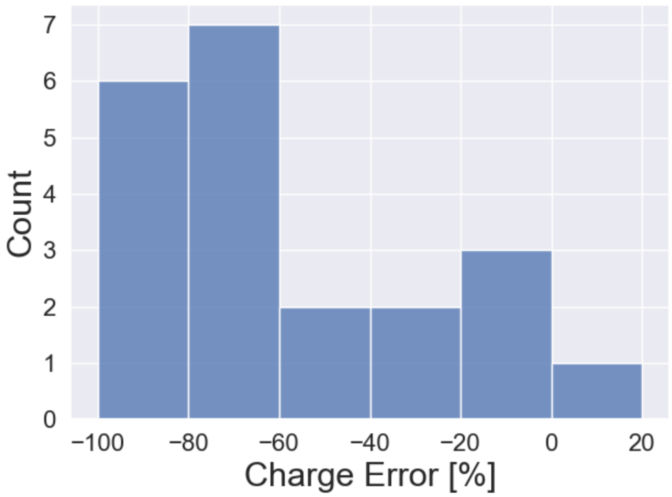

Fig. S15 Apparent charge error (believed to be influenced primarily by unintentional droplet dilution) across one set of preliminary demonstration reactions. The sole value in the [0,20] bin has value 0.2%

Buffer fluid*	Pre-run blow-dry?	Injection point	Conversion [%]	Charge error [%]
Gas	Yes	Withdraw into loop	51	+47
Gas	No	Withdraw into loop	41	+40
Oil	Yes	Withdraw into loop	45	-9
Gas	Yes	Injection valve	9	-64

Table S5 Conversion and limiting reagent charge error for a series of SNAr reactions performed at 0.002M 2,4-dichloroquinazoline, 80°C, 30min. *Oil buffer tests were coupled with 1.5x larger droplet preparation and twice the number of stirring operations.

12μL, a significant fraction of the original droplet volume.

Another phenomenon contributes to gradual evaporation of the droplet. Since the reactor tubing is heated and the temperature of the rest of the headspace is uncontrolled, there is a temperature gradient in the tubing outside the reactor, which creates a vapor pressure gradient along the tubing axis of any volatile compounds in the droplet. The vapor pressure gradient translates to a partial pressure gradient that serves as a driving force for diffusion from the zone where the vapor pressure is high (the heated tubing) to the zone where it is low (the exterior tubing). As vapor diffuses along this gradient, it becomes supersaturated and, as before, condenses.

The temperature gradient in the exterior tubing when the reactor is maintained at the maximum target operating temperature of 200°C was modeled using COMSOL Multiphysics software[§] (Figure S18). Based on the simulation results, the tubing outside the reactor cools very quickly with distance from the reactor, meaning vapor that is convected out of the reactor on each oscillation cycle contacts tubing that is at a much lower temperature, and that the temperature gradient that produces a vapor pressure gradient is steep.

To quantify evaporative losses, we monitored the change in the length of droplets over time, and computed the fraction of a droplet that evaporated by dividing the length of the droplet at a given time by its initial length. To allow for thermal expansion, we waited

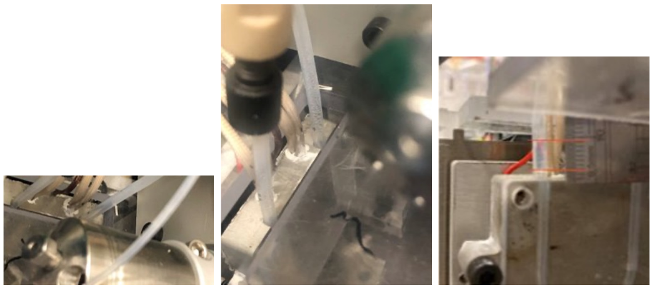

(a) (b) (c)

Fig. S16 Images of condensate forming outside heated reactor tubing

§ COMSOL Multiphysics® v. 5.6, www.comsol.com, COMSOL AB, Stockholm, Sweden

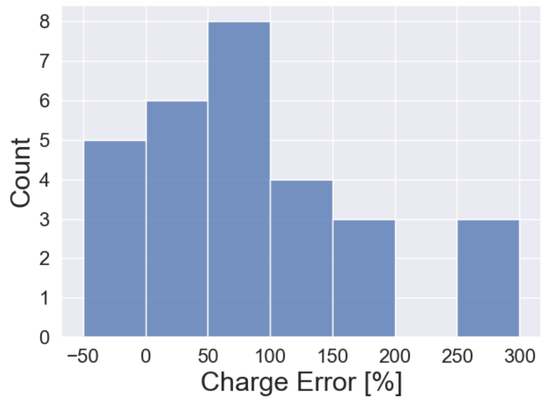

Fig. S17 Apparent charge error across preliminary demonstration reactions. The skew toward apparent overcharge is consistent with significant evaporation effects

Buffer fluid*	Pre-run vent?	Injection point	Conversion [%]	Charge error [%]
Gas	Yes	Withdraw into loop	51	+47
Gas	No	Withdraw into loop	41	+40
Oil	Yes	Withdraw into loop	45	-9
Gas	Yes	Injection valve	9	-64

Table S6 Conversion and limiting reagent charge error for a series of SNAr reactions performed at 0.002M 2,4-dichloroquinazoline, 80°C, 30min. *Oil buffer tests were coupled with 1.5x larger droplet preparation and twice the number of stirring operations.

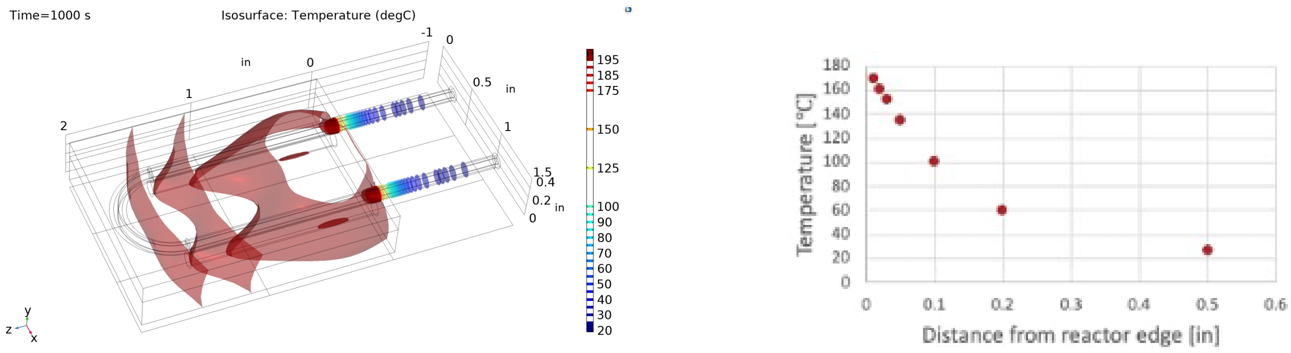

(a) COMSOL Multiphysics® simulation of temperature profile in the reactor and tubing outside it

(b) Temperature of the interior FEP tubing surface versus distance from reactor edge

Fig. S18 Results of COMSOL Multiphysics® simulation of reactor temperature profile

several seconds before collecting the t=0 length measurement in each case. To get an accurate measurement of droplet length, the oscillation was paused and droplets were pushed into a straight portion of the reactor tubing (Figure S19). Using a standard ruler, our detection limit is 1/64 of an inch, which translates to 0.8μL, or about 2% of the volume of a typical droplet. To compare the influence of different operating conditions and mitigation strategies, droplets of THF oscillating at 60°C for fifteen to thirty minutes were used. The saturation condition of the oscillator loop was also controlled: conditions in which the oscillator loop was pre-saturated with THF at 60°C were compared to those in which the oscillator loop was flushed before the experiment to remove THF from the vapor phase.

Several strategies for mitigating droplet evaporation were explored: we tried minimizing the total volume of the headspace, improving the condensing capabilities of the tubing outside the reactor by attaching a Peltier cooler to the tubing, and placing sacrificial droplets of pure solvent upstream and downstream of the main reaction droplet; none of these strategies reproducibly reduced evaporative losses to an acceptable level.

We eventually decided to examine the influence of removing oscillation from the design. Stationary droplets don't experience the

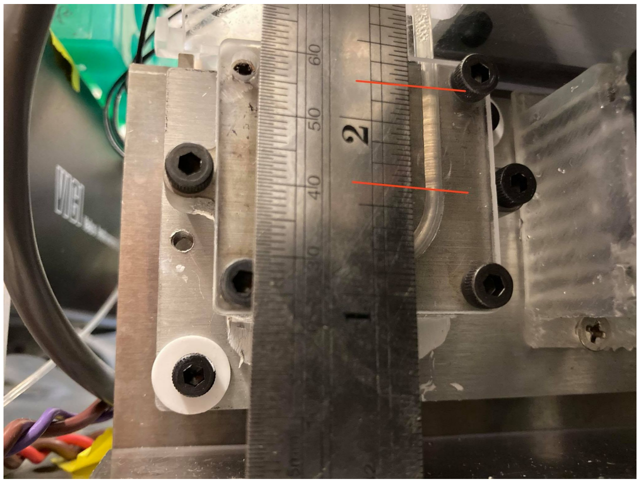

Fig. S19 Example of a droplet being measured to assess evaporation

convective contribution to evaporative losses described above, and the removal of the magnetic oscillator means the headspace can be shrunken significantly, from ∼5mL to ∼50μL. We performed a series of preliminary experiments examining the evaporation of stationary droplets in the same reactor used for the oscillation. In these experiments, stationary operation did reduce evaporative losses relative to the base case.

To further examine a stationary approach, we redesigned the reactor to use 0.02" ID tubing instead of the 0.0625" ID used in the oscillatory design, which reduces the interfacial area between the gas and the liquid and therefore slows evaporative behavior. To determine an appropriate tubing volume for the stationary reactor, thermal expansion coefficients of typical organic solvents were used to anticipate the change in droplet volume that would occur in response to a 175-degree temperature rise (from room temperature to our maximum target operating temperature of 200°C). Most organic solvents have thermal expansion coefficients in the range 0.001 to 0.002°C^{-1}, meaning that the reactor should be able to accommodate droplets up to volumes of around 54μL (Equation S2). We also wanted to ensure that the reactor would be able to accommodate the pressure resulting from carbon dioxide evolution from a 0.1M decarboxylative cross-coupling. Under worst-case conditions, the reaction would be operating at 200°C and the entire headspace would be at that temperature (in reality, tubing outside the reactor quickly cools to room temperature as distance from the reactor increases). At 200°C, the vapor pressure of a typical reaction solvent, THF, is around 300psi, and the nitrogen pressure in the system is 100psi. The pressure limit of the system is 500psi, so the additional headspace volume must be large enough to accommodate a 100psi pressure increase ($\approx 7\times10^5$Pa), which corresponds to a headspace volume of about 30μL (Equation S3).

$$\left(40\mu\text{L}\right)\left(175^\circ\text{C}\right)\left(0.002^\circ\text{C}^{-1}\right) = 54\mu\text{L} \tag{S2}$$

$$\begin{aligned} V \approx \frac{nRT}{P} &= \frac{\left(0.1\text{M}\right)\left(10^{-6}\text{L}/\mu\text{L}\right)\left(54\mu\text{L}\right)\left(473.2\text{K}\right)\left(8.314\text{m}^3\text{Pa/mol K}\right)}{\left(7\times10^5\text{Pa}\right)} \\ &= 3.03\times10^{-8}\text{m}^3 \approx 30\mu\text{L} \end{aligned} \tag{S3}$$

The updated stationary reactor that accounts for these conditions with safety factors has a heated volume of 60μL and connects to the six-port valve via eight total inches of temperature-uncontrolled 0.02" ID FEP tubing, which translates to a volume of about 40μL. The reactor was machined by ProtoLabs.

In the stationary reactor, we continued to use the change in droplet length over time to assess evaporative losses. The smaller diameter of the tubing makes the droplets harder to see, so small amounts of dye (Sudan red [Sigma-Aldrich, Sudan Red 7B, 95%] in THF [Sigma-Aldrich, anhydrous, ≥ 99.9%] or methylene blue [Sigma-Aldrich] in isopropanol [Sigma-Aldrich, anhydrous, 99.5%]) were added to the solvent to facilitate visualization. Neither dye was stable above 150°C, so stationary evaporation experiments above 150°C were not conducted.

We monitored evaporative losses from stationary droplets by setting the oscillation frequency to zero in the control software. At a temperature of 60°C (the same temperature at which most of the mitigation experiments described above were performed), no measurable evaporation had occurred after thirty minutes. Under the same conditions with oscillation, more than half of the droplet would typically evaporate. We further studied the effects of different temperatures and droplet sizes (Figure S20). Below 100°C, no evaporation was observed. Larger droplets experienced less evaporation, in a relative sense, than smaller ones.

The promising results obtained with stationary droplets in the oscillatory reactor setup motivated us to redesign the reactor so

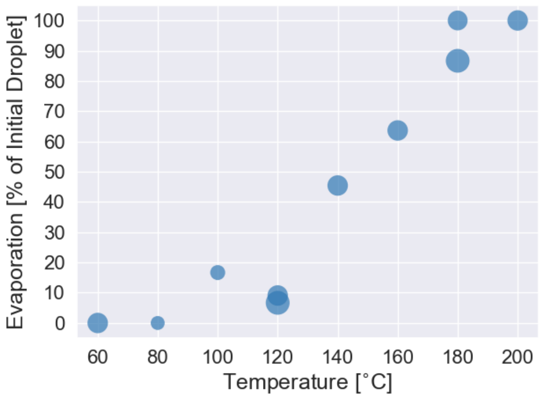

Fig. S20 Evaporative losses from stationary droplets heated for t=30min in the original oscillatory reactor. Point sizes reflect the relative initial sizes of the droplets

	Evaporative loss [%]		
Time [min]	100°C	125°C	150°C
5	0	0	0
15	0	0	8.6
30	0	0	8.6

Table S7 Evaporative losses from droplets of tetrahydrofuran (THF) in the stationary reactor at various timepoints and temperatures.

as to minimize the evaporation of stationary droplets. Results of evaporation monitoring of THF droplets at several timepoints and temperatures are shown in Table S7. The results are indicative of a significant reduction in droplet evaporation. As a result, we eliminated the magnetic oscillator from the design and made the updated stationary reactor a permanent fixture of the automated platform. Since the stationary droplets aren't actively mixed, the reactor system can no longer be used to study multiphase reactions.

S5 Bayesian reaction optimization with Dragonfly

S5.0.1 Dragonfly algorithm settings

- **Kernel:** the Matérn-2.5 kernel for Euclidean variables and the Hamming kernel for discrete categorical variables (Dragonfly default settings), see Kandasamy *et al.* for exact kernel definitions.

- **Initialization:** Latin hypercube sampling for Euclidean variables and uniform random sampling for discrete categorical variables (Dragonfly default). We varied the quantity of initialization experiments from 8 to 16, depending on the number of settings of the categorical variables being addressed in the case study in question.

- **Batch size:** We set the batch size, which is the quantity of experiments that the algorithm recommends on each iteration, to five, a value that balances between the execution efficiency gained through reaction parallelization and the trajectory efficiency that is naturally sacrificed when the algorithm is used to recommend several reactions at once (when there is more than one reaction in a batch, the reactions tend to be somewhat redundant).

The raw data from the two model reaction systems that we optimized using Dragonfly (Schemes 5 and 6) is included below.

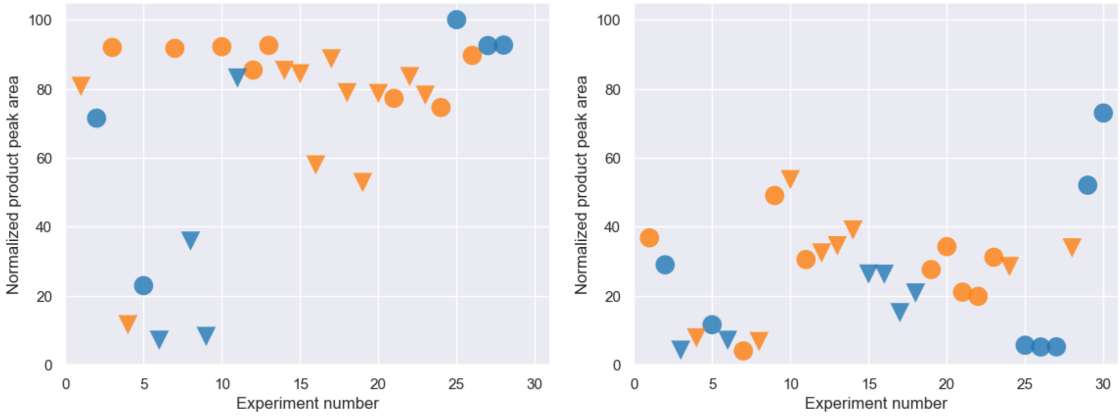

(a) Optimization trajectory in DMF. (b) Optimization trajectory in DMSO.

Fig. S21 Optimization trajectories for the reaction depicted in Scheme 5 in each of the investigated solvents. Marker shapes denote the identity of the base: circles for DBU and triangles for BTMG. Marker colors denote the identity of the catalyst: blue denotes tBuBrettPhos Pd G3 and orange denotes tBuXPhos Pd G3.

Exp #	Type	Temp [°C]	Time [min]	Prod. area [mAU s]	ISTD area [mAUs]	Product area (Norm.)	Cat	Base	Start mat. area [mAUs]	Start mat. area (Norm.)	Conversion [%]
1	initial	81.8	48.8	2344	5985	89.23	tBuXPhos	BTMG	32	1.2	98.9
2	initial	78.25	56.7	2126	6128	79.04	tBuBrettPhos	DBU	653	24.3	78.0
3	initial	97.1	10.9	2742	6141	101.72	tBuXPhos	DBU	11	0.4	99.6
4	initial	69.96	21.6	472	8376	12.84	tBuXPhos	BTMG	2737	74.4	32.4
5	initial	53.3	41.3	702	6329	25.27	tBuBrettPhos	DBU	2244	80.8	26.7
6	initial	62.6	19.11	226	6534	7.88	tBuBrettPhos	BTMG	1821	63.5	42.4
7	initial	87.1	29.1	3144.6	7064	101.42	tBuXPhos	DBU	47	1.5	98.6
8	initial	74.9	16.3	1113	6397	39.64	tBuBrettPhos	BTMG	1277	45.5	58.7
9	refine	51.2	60	296	7418	9.09	tBuBrettPhos	BTMG	2305	70.8	35.7
10	refine	75.3	21.2	2631	5880.5	101.93	tBuXPhos	DBU	62	2.4	97.8
11	refine	100	60	2519	6245	91.90	tBuBrettPhos	BTMG	37	1.3	98.8
12	refine	91.2	20.5	2700	6512	94.46	tBuXPhos	DBU	16.5	0.6	99.5
13	refine	85	20.9	2944	6552	102.37	tBuXPhos	DBU	32	1.1	99.0
14	refine	100	60	2788	6733	94.34	tBuXPhos	BTMG	31	1.0	99.0
15	refine	100	60	2755	6731	93.25	tBuXPhos	BTMG	21	0.7	99.4
16	refine	100	44.8	2690	9566	64.06	tBuXPhos	BTMG	1141	27.2	75.3
17	refine	100	45.3	2508	5827	98.06	tBuXPhos	BTMG	0	0.0	100.0
18	refine	100	52.8	2856	7462	87.20	tBuXPhos	BTMG	61	1.9	98.3
19	refine	90.1	49.2	2139	8345	58.40	tBuXPhos	BTMG	1549	42.3	61.6
20	refine	90	60	1474	3862	86.95	tBuXPhos	BTMG	0	0.0	100.0
21	refine	87.7	5	2273	6064	85.40	tBuXPhos	DBU	20	0.8	99.3
22	refine	90.4	11.2	2516	6206	92.36	tBuXPhos	BTMG	22	0.8	99.3
23	refine	90.2	26.6	2598	6850	86.41	tBuXPhos	BTMG	53	1.8	98.4
24	refine	100	60	3143	8685	82.45	tBuXPhos	DBU	1441	37.8	65.7
25	refine	100	60	4145	8537	110.62	tBuBrettPhos	DBU	53	1.4	98.7
26	refine	100	60	2071	4758	99.16	tBuXPhos	DBU	0	0.0	100.0
27	refine	100	53.3	2412	5371	102.31	tBuBrettPhos	DBU	0	0.0	100.0
28	refine	100	56.9	3027	6730	102.47	tBuBrettPhos	DBU	91	3.1	97.2

Fig. S22 Raw reaction optimization data from the optimization campaign of Scheme 5 performed in DMF.

Exp #	Type	Temp [°C]	Time [min]	Prod. area [mAU s]	ISTD area [mAU s]	Product area (Norm.)	Cat	Base	Start mat. area [mAU s]	Start mat. area (Norm.)	Conversion [%]
1	initial	95.9	8.9	1390	5534	40.69	tBuXPhos	DBU	1694	49.6	52.7
2	initial	92.3	36.9	1088	5500	32.05	tBuBrettPhos	DBU	1869	55.0	47.5
3	initial	58.9	27.7	158	5467	4.68	tBuBrettPhos	BTMG	1985	58.8	43.9
4	initial	72.5	50.1	295.6	5548	8.63	tBuXPhos	BTMG	1931	56.4	46.2
5	initial	81.3	59.4	461.8	5821	12.85	tBuBrettPhos	DBU	2327	64.8	38.2
6	initial	65.7	35.7	264	5450	7.85	tBuBrettPhos	BTMG	1979	58.8	43.9
7	initial	52.1	18.7	158	5743	4.46	tBuXPhos	DBU	2933	82.7	21.1
8	initial	55.7	36.4	250	5444	7.44	tBuXPhos	BTMG	2027	60.3	42.5
9	refine	100	55.5	1864	5565	54.26	tBuXPhos	DBU	1311	38.2	63.6
10	refine	100	5	2282	6232	59.32	tBuXPhos	BTMG	1193	31.0	70.4
11	refine	100	60	1137	5459	33.74	tBuXPhos	DBU	1570	46.6	55.6
12	refine	100	5	1188	5370	35.84	tBuXPhos	BTMG	1351	40.8	61.1
13	refine	100	9.1	1186	5037	38.14	tBuXPhos	BTMG	1211	38.9	62.9
14	refine	100	5	1365	5119	43.20	tBuXPhos	BTMG	1198	37.9	63.8
15	refine	91.7	48.8	870	4847	29.08	tBuBrettPhos	BTMG	1325	44.3	57.8
16	refine	100	60	989.6	5524	29.02	tBuBrettPhos	BTMG	1429.5	41.9	60.0
17	refine	91.8	28.8	525	5089	16.71	tBuBrettPhos	BTMG	1711	54.5	48.1
18	refine	91.7	5.6	748	5265	23.01	tBuBrettPhos	BTMG	1700	52.3	50.1
19	refine	100	59.1	1010	5360	30.53	tBuXPhos	DBU	1579	47.7	54.5
20	refine	100	60	1251	5350	37.88	tBuXPhos	DBU	1446	43.8	58.2
21	refine	89	12.6	724	5038	23.28	tBuXPhos	DBU	2077	66.8	36.3
22	refine	89.3	42.1	719	5312	21.93	tBuXPhos	DBU	1863	56.8	45.8
23	refine	100	5	1138	5346	34.48	tBuXPhos	DBU	1742	52.8	49.7
24	refine	100	60	1005	5174	31.47	tBuXPhos	BTMG	1391	43.6	58.5
25	refine	50	60	186	4802	6.27	tBuBrettPhos	DBU	2245	75.7	27.8
26	refine	50	52.6	185	5251	5.71	tBuBrettPhos	DBU	2546	78.5	25.1
27	refine	50	60	176	4951	5.76	tBuBrettPhos	DBU	2399	78.5	25.1
28	refine	100	8.4	1117	4829	37.47	tBuXPhos	BTMG	1211	40.6	61.3
29	refine	100	5	1781	5012	57.56	tBuBrettPhos	DBU	1160	37.5	64.2
30	refine	100	24.6	2211	4439	80.69	tBuBrettPhos	DBU	342	12.5	88.1

Fig. S23 Raw reaction optimization data from the optimization campaign of Scheme 5 performed in DMSO.

Exp #	Type	Temp [°C]	Time [min]	Prod. area [mAU s]	ISTD area [mAUs]	Product area (Norm.)	Cat	Base	Start mat. area [mAUs]	Start mat. area (Norm.)	Conversion [%]
1	initial	117.5	9.08	5469	1854	386.80	1	2	18	1.27	99.56
2	initial	96.4	24.4	4753	1925	323.76	1	1	788	53.68	81.38
3	initial	71.96	19.4	0	1827	0.00	2	1	3642	261.39	9.31
4	initial	84.6	8.1	5328	1795	389.21	1	2	89	6.50	97.74
5	initial	98.9	16.3	0	1861	0.00	2	1	3664	258.16	10.42
6	initial	108.2	13.2	6132	1966	408.98	1	1	0	0.00	100.00
7	initial	77.2	28.3	10	1792	0.73	2	2	3569	261.15	9.39
8	initial	78.9	10.5	17	1755	1.27	2	2	3522	263.15	8.70
9	refine	120	5	6008	1953	403.38	1	1	43	2.89	99.00
10	refine	106.8	5	6043	1965	403.25	1	1	0	0.00	100.00
11	refine	120	5	7198	2303	409.83	1	1	0	0.00	100.00
12	refine	119.3	5	6249	2007	408.27	1	1	0	0.00	100.00
13	refine	117.2	5	6535	2093	409.41	1	1	0	0.00	100.00
14	refine	120	5	6385	2028	412.84	1	1	0	0.00	100.00
15	refine	70	5	5629	1999	369.24	1	1	390.6	25.62	91.11
16	refine	74.2	5	6144	2036	395.69	1	1	147	9.47	96.72
17	refine	70.98	5.4	6409	2124	395.66	1	1	116	7.16	97.52
18	refine	70	5	6336	2034	408.46	1	1	26	1.68	99.42
19	refine	120	9.5	6663	2173	402.06	1	1	27	1.63	99.43
20	refine	120	9.6	6920	2207	411.14	1	1	0	0.00	100.00
21	refine	120	10.6	6761	2153	411.77	1	1	0	0.00	100.00
22	refine	120	10.8	7058	2217	417.45	1	1	0	0.00	100.00
23	refine	120	10	7136	2282	410.04	1	1	0	0.00	100.00
24	refine	120	5	6601	2180	397.04	1	2	21	1.26	99.56
25	refine	120	5	6918	2193	413.64	1	1	0	0.00	100.00
26	refine	120	5	6787	2204	403.79	1	2	0	0.00	100.00
27	refine	120	5	7428	2346	415.17	1	1	0	0.00	100.00
28	refine	120	5	7120	2263	412.55	1	1	0	0.00	100.00

Fig. S24 Raw reaction optimization data from the optimization campaign of Scheme 6.

S5.1 Kinetics investigation

The raw data from the kinetics demonstration campaign we performed is included in the table below.

Exp #	Temperature [°C]	C_0 FNB	Res. Time [min]	FNB Area [mAU]	ISTD Area [mAU]	C_FNB [M]
1	70	1.04	40	1610	4045	0.375
2	70	1.04	25	1938	3621	0.504
3	70	1.04	15	2453	3647	0.634
4	70	1.04	10	2981	4006	0.701
5	70	1.04	5	3358	3670	0.862
6	70	1.04	40	1907	4347	0.413
7	70	1.04	25	2032	3781	0.507
8	70	1.04	15	2622	3779	0.654
9	70	1.04	10	2880	3706	0.732
10	70	1.04	5	3592	4113	0.823
11	80	1.04	40	1192	3988	0.282
12	80	1.04	25	1665	3782	0.415
13	80	1.04	15	2207	3929	0.529
14	80	1.04	10	2509	3718	0.636
15	80	1.04	5	3206	3989	0.758
16	60	1.04	40	2172	4103	0.499
17	60	1.04	25	2424	3698	0.618
18	60	1.04	15	2802	3707	0.712
19	60	1.04	10	3246	3864	0.792
20	60	1.04	5	3593	3844	0.881
21	90	1.04	40	1244	4076	0.288
22	90	1.04	25	1426	3893	0.345
23	90	1.04	15	1856	3949	0.443
24	90	1.04	10	2295	3854	0.561
25	90	1.04	5	2898	3976	0.687
26	70	0.8	40	885	2947	0.218
27	70	0.8	25	1456	2922	0.361
28	70	0.8	15	1709	2705	0.458
29	70	0.8	10	2109	2819	0.542
30	70	0.8	5	2731	3108	0.637

Fig. S25 Raw data from kinetics investigation.
